# Dietary intake and cancer incidence in Korean adults: a systematic review and meta-analysis of observational studies

**DOI:** 10.4178/epih.e2023102

**Published:** 2023-11-30

**Authors:** Ji Hyun Kim, Shinyoung Jun, Jeongseon Kim

**Affiliations:** 1 National Cancer Center Graduate School of Cancer Science and Policy, National Cancer Center, Goyang, Korea; 2 Department of Food Science and Nutrition, Soonchunhyang University, Asan, Korea

**Keywords:** Diet, Neoplasms, Incidence, Korea, Systematic review, Meta-analysis

## Abstract

Cancer is a major health burden in Korea, and dietary factors have been suggested as putative risk factors for cancer development at various sites. This study systematically reviewed the published literature investigating the associations between dietary factors and cancer incidence among Korean adults, following the Preferred Reporting Items for Systematic Reviews and Meta-Analyses guidelines. We focused on the 5 most studied cancer sites (stomach, colorectum, breast, thyroid, and cervix) as outcomes and dietary exposures with evidence levels greater than limited-suggestive according to the World Cancer Research Fund/American Institute for Cancer Research (WCRF/AICR) panel’s judgment for any of the cancer sites. This resulted in the inclusion of 72 studies. Pooled estimates of the impact of dietary factors on cancer risk suggested protective associations of fruits and vegetables with risks for gastric cancer (GC), colorectal cancer (CRC), and breast cancer (BC) and dietary vitamin C with the risk of GC, as well as a harmful association between fermented soy products and the risk of GC. Despite the limited number of studies, we observed consistent protective associations of dietary fiber with GC and dietary fiber, coffee, and calcium with CRC. These findings are largely consistent with the WCRF/AICR expert report. However, pooled estimates for the associations of other salt-preserved foods with GC, meat with CRC, and dietary carotenoids and dairy products with BC did not reach statistical significance. Further studies with prospective designs, larger sample sizes, and diverse types of dietary factors and cancer sites are necessary.

## INTRODUCTION

Cancer remains a leading cause of death, with the number of newly diagnosed cases continuing to increase. Some types of cancer have shown only marginal improvements in patient survival outcomes, both globally and in Korea [1,2]. Therefore, a comprehensive primary prevention strategy for cancer should be implemented to reflect changing cancer statistics [1,3].

The elevated cancer burden may partially reflect increased life expectancy (i.e., population aging), which is a non-modifiable risk factor [4]. However, overwhelming evidence indicates that cancer pathogenesis is in part attributable to modifiable risk factors such as diet, smoking, alcohol consumption, physical inactivity, obesity, and environmental pollutants, suggesting that there is substantial potential for preventing cancer by targeting these factors [1,5-7].

The World Cancer Research Fund/American Institute for Cancer Research (WCRF/AICR) Continuous Update Project (CUP) provided some evidence for global-scale nutritional guidelines to reduce the risk of cancer at several anatomical sites [8]. However, although numerous epidemiological studies have been conducted during the last several decades, inconsistent findings have been reported regarding many dietary components’ effects on cancer, and the cumulative evidence is insufficient to draw robust conclusions [8,9]. Moreover, some of the currently available guidelines may not be generalized to diverse populations with distinct dietary behaviors and food preparation methods (e.g., cooking, fermenting, or using condiments).

Therefore, we aimed to summarize the evidence from observational studies on the relationship between diet and cancer in Korean adults, with the goal of informing nutritional guidelines for cancer prevention and identifying research gaps. We focused on cancers of the stomach, colorectum, breast, thyroid, and cervix, which were the most widely studied anatomical sites in relation to dietary factors in Korea. The dietary exposures were selected based on the WCRF/AICR panel’s judgment, which classified the evidence level as convincing, probable, or limited-suggestive regarding risk or protective factors for any of the cancer sites.

## MATERIALS AND METHODS

### Literature search strategy

We performed a comprehensive literature search of studies published between January 1, 2000 and December 31, 2022, in PubMed, Embase, and KoreaMed. The key search strategy included the following terms: (“diet” OR “food” OR “intake” OR “nutrition”) AND (“cancer” AND “risk”) AND (“Korea” OR “Korean”). Broad and non-specific terms such as “nutrition” were used to increase the likelihood of capturing potentially eligible studies. Articles published in English and Korean were considered. The detailed search terminologies used for each electronic database are available in Supplementary Material 1

### Literature search and study selection

**Figure 1.**
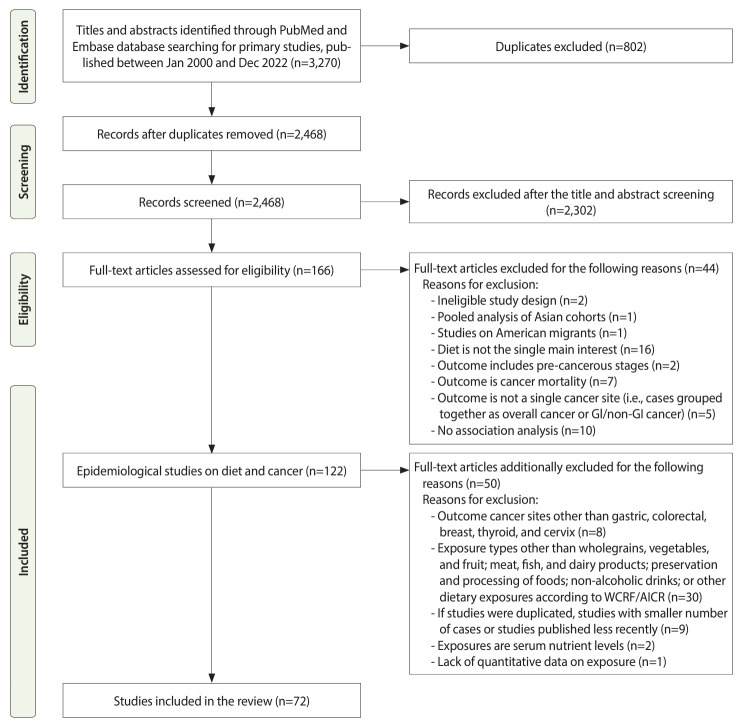
Preferred Reporting Items for Systematic Reviews and Meta-Analyses flow diagram of study selection, inclusion, and exclusion. GI, gastrointestinal; WCRF/AICR, World Cancer Research Fund/American Institute for Cancer Research.

This systematic review followed the Preferred Reporting Items for Systematic Review and Meta-Analyses guidelines (Figure 1).

The primary inclusion criteria were as follows: (1) study subjects: Korean adults residing in Korea; (2) study design: observational studies (cohort, case-control, and cross-sectional); (3) exposure: any exposure related to dietary factors other than exposures that explicitly examined alcohol or food contaminants that can lead to the addition or generation of potential carcinogens, such as aflatoxin (e.g., food/nutrient intake and dietary patterns); and (4) outcome: cancer incidence, not recurrence or mortality.

We further narrowed the scope with the following additional criteria: (1) exposure of interest: dietary factors that have been reported as convincing, probable, or limited-suggestive risk or protective factors for any of the cancer sites according to the WCRF/AICR CUP panel’s judgment (whole grains, fruits, and vegetables; meat, fish, and dairy products; preservation and processing of foods; non-alcoholic drinks; and other dietary exposures) [8] (Supplementary Material 2); (2) outcome of interest: the 5 most frequently studied cancer sites in the Korean population (stomach, colorectum, breast, thyroid, and cervix); and (3) if studies were duplicated (e.g., study participants, exposure dietary variables, and outcome cancer sites overlapped), we included studies with the following priority: (1) studies with a larger number of cases and (2) studies more recently published.

The titles, abstracts, and full-texts of all the retrieved references were independently reviewed by 2 researchers (JHK and SJ), and any potential disagreements were solved through consensus or the involvement of a third researcher (JK).

### Data extraction and quality assessment

The following data were extracted from the original publications: (1) first author and year of publication; (2) cancer site; (3) specified food items; (4) study design, enrollment year, and follow-up duration (only for cohort studies); (5) sample sizes: number of cases and number of controls for case-control studies, or number of non-cases for cross-sectional and cohort studies; (6) dietary assessment methods and dietary intake, specified as amount or frequency; and (7) main results: odds ratios (ORs) with 95% confidence intervals (CIs) for case-control or cross-sectional studies and relative risks (RRs) or hazard ratios (HRs) with 95% CIs for cohort studies.

For quality assessment and evaluation of the risk of bias, we used the Joanna Briggs Institute Critical Appraisal Tool for Systematic Reviews [10]. The assessed methodological criteria included 11, 10, and 8 items for cohort, case-control, and cross-sectional studies, respectively. Each of them was evaluated with 4 possible answers: “yes” (criterion met), “no” (criterion not met), “unclear,” and “not applicable (N/A).” If studies had average quality scores above 0.75 (75%), they were considered “high-quality,” whereas studies with quality scores lower than 0.75 were evaluated as “low-quality.” Additionally, for each criterion, a score was calculated by dividing the number of studies with positive scores by the total number of included studies to identify how well the current literature followed the criterion.

Data extraction and quality assessment were independently performed by 2 researchers (JHK and SJ), and inconsistencies were resolved by discussion or the involvement of a third researcher (JK).

### Data synthesis and statistical analyses

The evidence was summarized qualitatively for each of the dietary exposure groups and the cancer sites. The results are shown by the type of cancer and dietary factor classified according to the WCRF/AICR CUP panel’s judgment [8]. The information extracted from each study included dietary factors, study design, enrollment year, duration of follow-up, sample size, dietary assessment method, multivariable-adjusted risk estimates with CIs (except for 3 studies with crude values only), and data sources. The most common covariates were demographic factors (age and gender), socioeconomic factors (education and income), lifestyle factors (smoking, drinking, and physical activity), and family history of cancer; additionally, *Helicobacter pylori* infection was controlled for studies on gastric cancer (GC), and reproductive-related or hormone-related factors were controlled for breast cancer (BC) and cervical cancer (CC) (Supplementary Materials 3-7).

If 4 or more studies were available on the association between each dietary factor and cancer type, meta-analysis was performed to calculate the pooled risk estimate with a 95% CI. Heterogeneity was examined using the Higgins statistic (I^2^), which measures the percentage of variability across studies. Based on the heterogeneity of the included studies, fixed-effects or random-effects models were selected to calculate the pooled effect measures: when I^2^ was greater than 50% (substantial heterogeneity), the calculation was based on a random-effects model using the DerSimonian-Laird method, whereas if I^2^ was lower than 50%, the calculation was based on a fixed-effect model using the Woolf method. We also examined publication bias by using Begg’s funnel plots and Egger’s test: an asymmetric Begg’s funnel plot or a p-value < 0.05 in the Egger’s test were regarded as indicating publication bias. All analyses were performed using Stata SE version 14.0 (StataCorp., College Station, TX, USA).

### Ethics statement

Ethical approval was not sought because this study was based on published articles, and no human or animal intervention was performed.

## RESULTS

### Literature search

In total, 3,270 potential references were retrieved, 646 of which were in the PubMed database, 2,308 in the Embase database, and 316 in the KoreaMed database. After duplicates were removed, 2,468 references were screened by their titles and abstracts, and 2,302 references were excluded. The full-texts of the remaining 166 articles were assessed for eligibility, and 94 articles were subsequently excluded based on the aforementioned exclusion criteria. Finally, a total of 72 articles were included and reviewed systematically. The detailed study selection, inclusion, and exclusion processes are described in Figure 1.

### Characteristics and quality of the selected studies

The most common cancer sites studied were the stomach (25/72) and colorectum (24/72), followed by breast (20/72), thyroid (5/72), and cervix (4/72), with some studies exploring multiple cancer sites. The year of study participant enrollment ranged from 1993 to 2016, and the sample sizes ranged from 4,513 to 2,248,129 participants for cohort studies, 155 to 3,688 participants for case-control studies, and 56,934 to 162,220 participants for cross-sectional studies. The mean age of the study participants ranged from 48.4 years to 58.1 years for cohort studies, 44.2 years to 59.6 years for case-control studies, and 53.2 years to 53.6 years for cross-sectional studies. Except for studies conducted focused on a specific gender, the proportion of men ranged from 33.8% to 70.2%. Most of the case-control studies recruited both cases and controls from primary health clinics or hospitals, while some studies used community controls. All cohort studies identified newly diagnosed cases from cancer registries. All cross-sectional studies used questionnaire-based medical histories.

Among 72 studies that entered the review, 10 out of 10 cohort studies, 53 out of 60 case-control studies, and 2 out of 2 cross-sectional studies were evaluated as high-quality studies according to the assessment tool for systematic reviews from the Joanna Briggs Institute [10]. Supplementary Materials 8-13 present the percentage of studies meeting the quality criteria and provide detailed information on the quality score of each study.

### By cancer type

#### Gastric cancer

We identified 25 studies on dietary intake and GC (Table 1) [11-35].

##### Whole grains, fruits, and vegetables

Both case-control studies on dietary fiber showed significant inverse associations with the risk of GC [11,12]. Among 6 studies on fruits and vegetables, significant inverse associations with the risk of GC were observed in 3 case-control studies for fruits [15], both fruits and vegetables [17], and green vegetables [19]; however, no significant associations were observed in 2 cohort studies [13,14] and 2 case-control studies [16,18]. Three case-control studies assessed dietary carotenoid classes on GC risk. Two studies observed protective effects of lycopene [21] and β-carotene [12] on the risk of GC, while another study identified a non-significant effect of β-carotene [20].

##### Meat, fish, and dairy products

There were 3 studies on red meat and GC risk. A cohort study on red meat showed a non-significant association [13], whereas 2 case-control studies showed inconsistent results: a study investigating different types of red meat identified an increased risk associated with charcoal grilled beef [18], and another study found that cooked beef was associated with a reduced risk of GC [25].

##### Preservation and processing of foods

Among 7 studies on pickled vegetables, 4 case-control studies observed an elevated risk of GC, with at least 1 food item classified as kimchi [17,18,26,28]; however, no significant associations of pickled vegetables or kimchi with GC were observed in 1 cohort study [30] and 2 case-control studies [15,29]. Among fermented soy products, 2 case-control studies observed an increased risk of GC associated with soybean paste or stew [19,28], whereas a cohort study [14] and a case-control study [23] showed that the associations were non-significant. With regard to salted seafood and fish, non-significant associations with GC risk were observed in a cohort study [30] and a case-control study [18], while the associations were inconsistent in 2 case-control studies, which reported borderline increased [17] or decreased risk [26]. Among 3 studies on sodium intake, increased GC risk was observed in 2 studies (a cohort study [13] and 1 case-control study [28]), while another case-control study showed a non-significant association [12].

##### Other dietary exposures

Two case-control studies showed protective effects of plant-based dietary patterns on GC risk: a pattern termed the “prudent diet” derived from factor analysis with high loadings of fruits, vegetables, and other plant foods (e.g., tubers, mushrooms, tofu/soymilk, and nuts) [32] and index-based patterns on dietary antioxidant capacity, which were estimated based on the oxygen radical absorbance capacity and mainly comprised fruits and vegetables [33].

#### Colorectal cancer

We identified 24 studies on dietary intake and colorectal cancer (CRC) (Table 2) [13,31,36-57].

##### Whole grains, fruits, and vegetables

Both case-control studies on dietary fiber and CRC risk showed significant inverse associations [36,37]. For 6 studies on fruits and vegetables, significant inverse associations with CRC risk were observed in 3 case-control studies: 1 for fruits and vegetables combined and vegetables [38], 1 for both separate groups of fruits and vegetables [37], and 1 for a group composed of banana, pear, apple, and watermelon considered protective only for men [39]; however, non-significant associations were observed in a cohort study [13] and 2 case-control studies [36,40]. There were 3 case-control studies on dietary carotenoid classes: protective effects were observed for lutein/zeaxanthin [41] and carotene [37], whereas the association was non-significant for β-carotene [36].

##### Meat, fish, and dairy products

With regard to meat intake, 1 cohort study [43] and 1 case-control study [40] showed that more frequent meat consumption was associated with an elevated risk of CRC; however, another case-control study did not find any significant association [44]. The results regarding red meat and CRC risk were inconclusive, especially for case-control studies: 1 for a decreased risk [45], 1 for an elevated risk [36], and 2 non-significant associations [37,46]. One cohort study on red meat also showed a non-significant association with CRC risk [13]. Among 3 case-control studies on milk and dairy products, 2 reported an increased risk of CRC [36,45], whereas another study identified a protective effect of milk on CRC [37]. Three studies investigated the association between dietary calcium and CRC risk; 2 case-control studies found inverse associations with CRC risk [37,48], whereas the association was non-significant in a cohort study [47].

##### Non-alcoholic drinks

A case-control study [50] and a cross-sectional study [31] showed that coffee consumption had a protective effect on CRC.

##### Other dietary exposures

Saturated fatty acids were found to have significant associations with CRC risk, but the direction of associations differed across studies, with 1 study reporting a positive association [36] and 1 study reporting an inverse association [37]. Five case-control studies showed that dietary patterns highly correlated with inflammatory or insulinemic potential may significantly increase the risk of CRC. Among the studies with statistically derived patterns, one applied reduced rank regression using food groups as predictors and the plasma C-reactive protein (CRP) concentration as a response and derived the CRP-dietary pattern score, which showed inverse correlations with fruits and vegetables [54]. In another study, a pattern termed the “Westernized diet” was derived from factor analysis with high loadings of meats (red meat, meat byproducts, and poultry), high-carbohydrate foods, and oil [55]. Studies with index-based patterns were defined based on prior evidence, including components selected a priori based on the previous literature and biological plausibility. The procedure of assessing the dietary inflammatory index involved calculating scores of food parameters for inflammation based on a weighting algorithm to account for the robustness of evidence [56]. The dietary inflammation score method jointly assessed inflammation-related dietary factors by weighing each component based on its association with inflammatory biomarkers [52]. Additionally, given that cumulative evidence has suggested mechanical linkages between insulin levels and colorectal carcinogenesis, the insulinemic potential of diets (empirical dietary indices for hyperinsulinemia and insulin resistance) was calculated by utilizing indices based on food groups contributing to hyperinsulinemia (C-peptide) and insulin resistance (triacylglycerol: high-density lipoprotein cholesterol) and was consequently weighted by the regression coefficients [53].

#### Breast cancer

We identified 20 studies on dietary intake and BC (Table 3) [31,58-76].

##### Whole grains, fruits, and vegetables

In 6 studies on fruits and vegetables, significant inverse associations with BC risk were observed in 3 case-control studies for combined fruits and vegetables, vegetables, and non-pickled vegetables [61], fruits [63], and fruits and green vegetables, but not for white vegetables [64]. Non-significant associations were observed in a cohort study [60] and a case-control study [62].

##### Meat, fish, and dairy products

For any type of meat, an elevated risk of BC was observed in a cohort study of grilled ribs or barbecue [60] and in a case-control study on meat, including beef, pork, and chicken [64]; however, the other 2 case-control studies did not find any significance [59,63]. The results were conflicting among 5 studies on fish: significant protective associations with the risk of BC were found in a case-control study on total and fatty fish [70], whereas another case-control study indicated an increased risk of BC among those who consumed total fish (any kind) more frequently [64]. However, a cohort study [60] and 2 case-control studies [59,63] did not report any significant findings.

##### Other dietary exposures

The glycemic index showed significant associations with BC risk, but the direction of associations differed across studies: 1 for an increased risk [74] and 1 for a reduced risk [76]. One cohort study and 2 case-control studies showed that dietary patterns highly correlated with inflammatory or glycemic responses may significantly increase the risk of BC. With regard to inflammation, there was a study on an index-based dietary inflammatory index [73]. Additionally, in studies with statistically derived patterns, a pattern termed the “white rice diet” was derived from factor analysis with high loadings of white rice and lower loadings of multigrain rice [72], and a study applied reduced rank regression using food groups as predictors and glycemic index or glycemic load as responses, where grain intake explained most of the variance in the factor scores in both glycemic patterns [74].

#### Thyroid cancer

We identified 5 studies on dietary intake and thyroid cancer (TC) (Table 4) [13,31,77-79]. Dietary calcium, coffee, higher adherence to noodle/meat pattern, and lower adherence to prudent pattern were associated with a reduced risk of TC; however, these results were found only in 1 study.

#### Cervical cancer

We identified 4 studies on dietary intake and CC (Table 5) [31,80-82]. Dietary vitamin C was associated with a decreased risk of CC, while the dietary inflammatory index was correlated with a borderline increased risk of CC. However, those results were based on only 1 study.

### Results of meta-analysis

Table 6 shows the results of a meta-analysis of the impact of dietary factors on cancer risk for exposure-outcome pairs with 4 or more observational studies. In this analysis, the consumption of fruits and vegetables (highest vs. lowest) was significantly associated with a decreased risk of GC, CRC, and BC in a random-effects model (GC: OR, 0.59; 95% CI, 0.40 to 0.86; I^2^= 82.2%; CRC: OR, 0.63; 95% CI, 0.49 to 0.80; I^2^= 51.4%; BC: OR, 0.72; 95% CI, 0.53 to 0.98; I^2^= 77.0%). Total fruit intake was associated with a reduced risk of CRC in a fixed-effect model (OR, 0.69; 95% CI, 0.56 to 0.86; I^2^= 23.2%) but not for GC and BC in a random-effects model. Total vegetable intake was inversely associated with GC and CRC in a random-effects model (GC: OR, 0.54; 95% CI, 0.32 to 0.90; I^2^= 84.6%; CRC: OR, 0.58; 95% CI, 0.42 to 0.80; I^2^= 62.4%) but was non-significant for BC in a fixed-effect model. Dietary vitamin C was also inversely associated with GC risk in the fixed-effect model (OR, 0.74; 95% CI, 0.59 to 0.92; I^2^= 0.0%), and fermented soy products were positively associated with GC risk in a random-effects model (OR, 1.56; 95% CI, 1.08 to 2.27; I^2^= 56.3%). For those results, except for the associations between salt-preserved vegetables and GC, no evidence of publication bias was observed. The Begg’s funnel plots were symmetric, and the p-values for bias using the Egger’s test were > 0.05 (Supplementary Materials 14-34).

## DISCUSSION

In this systematic review, we summarized relatively recent publications on dietary intake and the risks of major cancers among the Korean adult population. A substantial number of studies were published recently or conducted since the publication of previous review articles on diet and cancer among Koreans in 2011 and 2014 [83,84], including studies on TC [77-79]; CC [80,81]; cancer at diverse anatomical sites [31]; GC in relation to specific food items and nutrients [11,15,20-24,26,27,29,30], dietary pattern [32-34], and the glycemic index [35]; CRC in relation to specific food items and nutrients [36,41,42,45-51], dietary patterns [52-56], colors of foods [38], and the glycemic index [57]; and BC in relation to specific food items and nutrients [59,60,66,68,71], dietary patterns [72-75], and the glycemic index [74]. Some studies have also explored gene and diet interactions in GC [11,23,26,27,33] and CRC [41,42,45,46,53,54,57].

The pooled estimates of dietary factors on cancer risk suggested protective associations of fruits and vegetables with the risks of GC, CRC, and BC and of dietary vitamin C with that of GC, as well as a harmful association of fermented soy products with the risk of GC. In addition, despite the limited number of previous studies, we observed consistent trends for inverse associations of dietary fiber with GC risk and of dietary fiber, coffee, and calcium with CRC risk. The results were null or insufficient for other foods preserved by salting (vegetables and seafood/fish) and grilled meat/fish in relation to the risk of GC; red or processed meat, dairy products, fish, heme iron, and vitamin C and D in relation to the risk of CRC; and dietary carotenoids, dairy products, and calcium in relation to the risk of BC. We compared our findings with those from the most recent WCRF/AICR report [8] and discussed plausible mechanisms underlying each finding below.

### The impact of foods preserved by salting on gastric cancer

The findings of this review showed that fermented soybean paste containing a substantial amount of salt had a positive association with GC. This result is in line with the WCRF/AICR’s latest report, which concluded that there is probable evidence to support that foods preserved by salting may increase GC risk, because the increased intragastric sodium concentration can damage the stomach mucosal barrier, leading to atrophic gastritis and *H. pylori* colonization [8,85]. *H. pylori*-associated gastritis may increase endogenous nitrite synthesis and decrease intragastric vitamin C secretion, thereby increasing the formation of endogenous N-nitroso compounds [86,87]. Moreover, the processing and storage of vegetables or soy products under acidic or oxygenic conditions with greater amounts of salt may consequently lead to the loss of antioxidant nutrients [86,88].

### The impact of fruits or vegetables on gastric cancer, colorectal cancer, breast cancer, and dietary fiber/coffee on colorectal cancer

This review supports WCRF/AIRC’s probable evidence indicating that consuming greater amounts of foods containing dietary fiber may decrease the risk of CRC and the limited-suggestive evidence for fruits or vegetables and the risks of GC, CRC, and BC [8]. Fruits and vegetables are rich sources of bioactive compounds (e.g., vitamins such as vitamin C, minerals such as calcium, and phytochemicals such as carotenoids), and the variability of choices may further synergistically enhance the effects of constituents on molecular mechanisms through their antioxidant and anti-inflammatory properties, which trigger diverse signaling pathways to prevent cancer [89,90]. In addition to these health-promoting substances, plant foods also contain fiber, which can also shorten the intestinal transit time and dilute carcinogenic contents in the intestine [91]. The anaerobic fermentation of fiber in the intestine by gut bacteria produces short-chain fatty acids, which can stimulate the secretion of hormones (GLP-1, PYY) that assist glucose metabolism (e.g., increasing insulin secretion and controlling blood glucose levels), thereby playing a key role in cancer prevention [91]. Additionally, a beneficial effect of coffee on CRC was observed in this review, probably due to the antioxidant and anti-inflammatory properties of its phytochemicals (e.g., polyphenols and melanoidins), which protect against inflammation-triggered carcinogenesis [92]. However, the WCRF/AICR report contains limited or no conclusions regarding CRC and coffee consumption [8].

### The impact of meat intake on colorectal cancer

In this meta-analysis, non-significant results were observed for the effect of meat on CRC, probably due to the limited number of studies and the lower meat consumption in Korea compared to other countries. Nevertheless, several publications in this review suggested that consuming meat more frequently is a risk factor for CRC, although the exact types of meat were not clarified. Red and processed meat have been judged as probable and convincing risk factors according to the WCRF/AICR report and were classified as group 2A (probable carcinogen) and group 1 (carcinogen) for humans according to the International Agency for Research on Cancer, respectively [8,93]. When meat is cooked at high temperatures (e.g., grilling, barbecuing, panfrying muscle meat, or cooking over a direct flame) or processed (e.g., curing and smoking), carcinogenic chemicals such as polycyclic aromatic hydrocarbons and heterocyclic amines are formed, and they can play a key role in the pathogenesis of CRC through the increased production of DNA adducts [93]. The heme iron content of red and processed meat can catalyze the formation of N-nitroso compounds and can induce lipid peroxidation in intestinal epithelial cells, which may be responsible for gene alterations [94].

### The impact of dietary calcium and dairy products on colorectal cancer

To evaluate the quality of evidence, an umbrella review was conducted with meta-analyses from the WCRF/AICR report. From this evaluation, a strong association between calcium and a lower risk of CRC has been inferred by the strength and significance of results with less bias [95]. Concordant with this study, 2 out of 3 publications that we identified showed a reduced risk of CRC. Dietary calcium can directly affect cell proliferation and differentiation and participate in a cascade of intercellular connections and signal transduction, influencing cell cycle regulatory genes that are involved in colorectal carcinogenesis [96]. Additionally, dietary calcium can bind to bile acids in the intestinal lumen and form insoluble calcium soaps, which can further protect the mucous membrane from the cytotoxicity caused by fatty acids [96,97]. WCRF/AICR has reported a probable reduced risk of CRC development due to dairy products, which has been largely attributed to their calcium content [8,95]. However, our results showed inconclusive findings, as 1 study reported protective effects of dairy products on CRC, whereas 2 out of 3 studies showed increased CRC risk in individuals who consumed more dairy products.

We could not draw any conclusions from studies on TC and CC. This is not surprising because TC is not generally recognized to have a relationship with diet [8]. However, we included the results for TC because it is the most frequent cancer in Korea [2], with the aim of identifying any potential dietary factors linked to TC in the Korean population. Similarly, the WCRF/AICR systematic literature review on CC could not draw any evidence for dietary variables [8,98], but we included this site because it was 1 of the top 5 most frequently studied anatomical sites in relation to dietary factors in Korea.

Our review faced several challenges, some of which may be due to research gaps in diet-cancer epidemiological studies among Korean adults. First, specifying single dietary factors was challenging because foods were often grouped together (e.g., all types of meat grouped as “meat” instead of a specific distinction between red meat and other types of meat, and “total fruits and vegetables” instead of separate analyses of fruits and vegetables), especially for studies conducted earlier. Further studies disaggregating mixed dishes into component parts are warranted to better estimate the exact intake of specific food items. Moreover, large variations in dietary assessment tools (e.g., studies that use validated food frequency questionnaires vs. short-form questionnaires based on intake frequency) and discrepancies in study design and exposure classification made it difficult to compare different studies. Our findings should be interpreted cautiously because the majority of publications selected in this review had case-control (60/72) and cross-sectional (2/72) designs. Unless exposures remain stable over time and are not affected by the outcome, those designs are prone to bias (e.g., selection and information), leading to weaker evidence of causality compared to a cohort design. Last, some cancer sites with high incidence rates in Korea (e.g., prostate, lung, liver, and pancreas) have not been extensively studied in relation to diet. Based on these challenges, we proposed areas of focus for future epidemiological research on dietary intake and cancer risks in Korea (Table 7).

## CONCLUSION

This study reviewed the recent literature on the associations between dietary factors and cancer risks among Korean adults. By pooling the estimates of observational studies, we found protective associations of fruits and vegetables with GC, CRC, and BC risk and dietary vitamin C with GC risk, as well as a harmful association of fermented soy products with GC risk. In addition, despite limited numbers of studies, protective associations were observed between dietary fiber and GC risk as well as dietary fiber, coffee, and calcium with CRC risk. These findings are highly concordant with the expert report provided by the WCRF/AICR based on a series of meta-analyses at a global scale.

However, other findings of the present study did not fully support the WCRF/AICR report. In the current meta-analysis, nonsignificant associations were observed for pickled vegetables and salted seafood/fish with GC risk, red meat with CRC risk, and dietary carotenoids and dairy products with the risk of BC. Furthermore, this study identified insufficient evidence for the associations of grilled meat and fish with GC risk, processed meat, dairy products, fish, heme iron, and vitamins C and D with CRC risk, and dietary calcium with BC risk. Further studies focusing on the longitudinal designs, larger sample sizes, and diverse dietary factors with a comprehensive list of cancer types are warranted.

## Figures and Tables

**Table 1.** Literature review on diet and gastric cancer in Korea

Dietary factors	Study design, enrollment year, follow-up duration (yr)	Sample size (cases/controls, non-cases), age (yr), % of men	Diet assessment, amount or frequency	Risk estimate			Sources	Year [Ref]
				Category	Type	Effect (95% CI)		
Whole grains, fruits, and vegetables								
Dietary fiber (2 studies)								
Dietary fiber	Case-control, 2011-2014	377/756, mean age: 53.8, men: 65.6	106-item FFQ, amount	T3 vs. T1 (reference)	OR	0.37 (0.24, 0.57)	National Cancer Center	2022 [11]
Dietary fiber	Case-control, 1997-1998	136/136, mean age: 57.2, men: 68.4	109-item FFQ, amount	Q4 vs. Q1 (reference)	OR	0.37 (0.17, 0.79)	Hanyang and Hallym University Hospital	2005 [12]
Fruits and vegetables (7 studies)								
Fruits and vegetables	Cohort, 2004-2008, median follow-up: 7.0	46/7,637, mean age: 48.4, men: 54.8	3-day DR, amount	≥600 vs. <600 g/day (reference)	HR	0.83 (0.35, 1.98)	National Cancer Center	2014 [13]
Fruits	Cohort, 1993-2004, mean follow-up: 8.5	166/9,558, mean age: 57.6, men: 68	14-item brief FFQ, frequency	≥1 time/day vs. almost never (reference)	RR	1.10 (0.55, 2.22)	Korean Multi-Center Cancer Cohort	2013 [14]
Vegetables				≥1 time/day vs. almost never (reference)		0.68 (0.27, 1.68)		
Fruits	Case-control, 2011-2014	415/830, mean age: 53.7, men: 65.1	106-item FFQ, amount	T3 vs. T1 (reference)	OR	0.59 (0.41, 0.85)	National Cancer Center	2016 [15]
Vegetables				T3 vs. T1 (reference)		0.96 (0.68, 1.34)		
Fresh vegetables	Case-control, 1997-2003	421/632, mean age: 59.6, men: 65.5	84-item FFQ, amount	Upper vs. lower median (reference)	OR	0.92 (0.72, 1.17)	Chungbuk and Eulji University Hospital	2005 [16]
Fruits	Case-control, 1999	69/199, most frequent age range: 41-55, men: 61.9	161-item FFQ, frequency	>6 vs. <4/wk (reference)	OR	0.30 (0.10, 0.70)	Asan Medical Center	2003 [17]
Raw vegetables				>5 vs. <3/wk (reference)		0.20 (0.10, 0.50)		
Fruits	Case-control, 1997-1998	136/136, mean age: 57.2, men: 68.4	109-item FFQ, amount	Q4 vs. Q1 (reference)	OR	0.67 (0.33, 1.39)	Hanyang and Hallym University Hospital	2002 [18]
Vegetables						0.64 (0.31, 1.32)		
Green vegetables	Case-control, 1997-1999	204/204, mean age: 59.5, men: 68.8	84-item FFQ, frequency	≥1/wk vs. <1/mo (reference)	OR	0.24 (0.14, 0.41)	Chungbuk National University Hospital	2000 [19]
Dietary carotenoids (3 studies)								
Dietary β-carotene	Case-control, 2002-2006	286/286, mean age: 56.8, men: 66.4	102, 115-item FFQ, amount	Q4 vs. T1 (reference)	OR	0.97 (0.60, 1.56)	Hanyang and Chungnam National University Hospital	2022 [20]
Dietary total carotenoids	Case-control, 2011-2014	415/830, mean age: 53.7, men: 65.1	106-item FFQ, amount	T3 vs. T1 (reference)	OR	0.79 (0.55, 1.15)	National Cancer Center	2018 [21]
Dietary α-carotene				T3 vs. T1 (reference)		1.00 (0.70, 1.41)		
Dietary β-carotene				T3 vs. T1 (reference)		0.85 (0.59, 1.22)		
Dietary β-cryptoxanthin				T3 vs. T1 (reference)		0.77 (0.54, 1.10)		
Dietary lutein/zeaxanthin				T3 vs. T1 (reference)		0.91 (0.64, 1.30)		
Dietary lycopene				T3 vs. T1 (reference)		0.60 (0.42, 0.85)		
Dietary β-carotene	Case-control, 1997-1998	136/136, mean age: 57.2, men: 68.4	109-item FFQ, amount	Q4 vs. Q1 (reference)	OR	0.35 (0.16, 0.75)	Hanyang and Hallym University Hospital	2005 [12]
Dietary vitamin C (4 studies)								
Dietary vitamin C	Case-control, 2002-2006	286/286, mean age: 56.8, men: 66.4	102, 115-item FFQ, amount	Q4 vs. T1 (reference)	OR	0.84 (0.52, 1.36)	Hanyang and Chungnam National University Hospital	2022 [20]
Dietary vitamin C	Case-control, 2011-2014	415/830, mean age: 53.7, men: 65.1	106-item FFQ, amount	T3 vs. T1 (reference)	OR	0.71 (0.50, 1.00)	National Cancer Center	2016 [15]
Dietary vitamin C	Case-control, 1997-1998	136/136, mean age: 57.2, men: 68.4	109-item FFQ, amount	Q4 vs. Q1 (reference)	OR	0.55 (0.27, 1.12)	Hanyang and Hallym University Hospital	2005 [12]
Dietary vitamin C	Case-control, 1997-1998	295/295, mean age: 49.3, men: 70.2	84-item FFQ, amount	>93.3 vs. ≤93.3 mg/day (reference)	OR	0.79 (0.52, 1.21)	Seoul National University Hospital and Asan Medical Center	2005 [22]
Dietary isoflavone (1 study)								
Dietary isoflavone	Case-control, 2011-2014	377/754, mean age: 53.8, men: 65.3	106-item FFQ, amount	T3 vs. T1 (reference)	OR	0.70 (0.49, 1.00)	National Cancer Center	2017 [23]
Meat, fish, and dairy products								
Meat (5 studies)								
Meat	Cohort, 1993-2004, mean follow-up: 8.5	166/9,558, mean age: 57.6, men: 68.4	14-item brief FFQ, frequency	≥1 time/day vs. almost never (reference)	RR	0.88 (0.30, 2.60)	Korean Multi-Center Cancer Cohort	2013 [14]
Meat	Cohort, 1996-1997, follow-up: 6-7	12,393/2,235,736, most frequent age range: 50-59, men: 63.2	A single question, frequency	≥4 vs. ≤1/wk	HR	0.99 (0.93, 1.07)	Korean Health Insurance Cooperation	2010 [24]
Red meat	Cohort, 2004-2008, median follow-up: 7.0	46/7,637, mean age: 48.4, men: 54.8	3-day DR, amount	≥600 vs. <600 g/day (reference)	HR	1.16 (0.56, 2.41)	National Cancer Center	2014 [13]
Total beef	Case-control, 1997-1998	136/136, mean age: 57.2, men: 68.4	109-item FFQ, amount	Q4 vs. Q1 (reference)	OR	1.67 (0.86, 3.27)	Hanyang and Hallym University Hospital	2002 [18]
Total pork				Q4 vs. Q1 (reference)		0.94 (0.45, 1.97)		
Cooked beef	Case-control, 2000	69/199, most frequent age range: 41-55, men: 61.9	161-item FFQ, frequency	≥1 vs. <1/mo (reference)	OR	0.40 (0.20, 0.80)	Asan Medical Center	2002 [25]
Grilled meat and fish (2 studies)								
Fried meat and fish	Case-control, 1997-1998	136/136, mean age: 57.2, men: 68.4	109-item FFQ, amount	Q4 vs. Q1 (reference)	OR	0.73 (0.36, 1.48)	Hanyang and Hallym University Hospital	2002 [18]
Charcoal grilled beef	Case-control, 1997-1998	136/136, mean age: 57.2, men: 68.4	109-item FFQ, amount	Q4 vs. Q1 (reference)	OR	2.11 (1.17, 3.82)	Hanyang and Hallym University Hospital	2002 [18]
Charcoal grilled beef and pork				Q4 vs. Q1 (reference)		1.58 (0.80, 3.10)		
Fish (3 studies)								
Fresh fish	Cohort, 1993-2004, mean follow-up: 8.5	166/9,558, mean age: 57.6, men: 68.4	14-item brief FFQ, frequency	≥1 time/day vs. almost never (reference)	RR	1.46 (0.65, 3.28)	Korean Multi-Center Cancer Cohort	2013 [14]
Raw fish	Case-control, 1997-2001	214/214, mean age: 58.8, men: 67.5	89-item FFQ, amount	Upper vs. lower median (reference)	OR	0.68 (0.46, 1.01)	Chungbuk National and Eulji University Hospital	2003 [26]
Slices of raw fish	Case-control, 1997-1999	204/204, mean age: 59.5, men: 68.8	84-item FFQ, frequency	≥1/wk vs. <1/mo (reference)	OR	0.43 (0.04, 4.81)	Chungbuk National University Hospital	2000 [19]
Dairy products (2 studies)								
Dairy product	Cohort, 1993-2004, mean follow-up: 8.5	166/9,558, mean age: 57.6, men: 68.4	14-item brief FFQ, frequency	≥1 time/day vs. almost never (reference)	RR	1.30 (0.83, 2.06)	Korean Multi-Center Cancer Cohort	2013 [14]
Dairy product	Case-control, 1997-1998	136/136, mean age: 57.2, men: 68.4	109-item FFQ, amount	Q4 vs. Q1 (reference)	OR	0.68 (0.34, 1.36)	Hanyang and Hallym University Hospital	2002 [18]
Dietary iron (3 studies)								
Dietary total iron	Case-control, 2011-2014	374/754, mean age: 53.8, men: 65.6	106-item FFQ, amount	T3 vs. T1 (reference)	OR	0.65 (0.45, 0.94)	National Cancer Center	2021 [27]
Dietary non-heme iron				T3 vs. T1 (reference)		0.64 (0.44, 0.92)		
Dietary heme iron				T3 vs. T1 (reference)		0.81 (0.56, 1.17)		
Dietary iron	Case-control, 2000-2005	471/471, mean age: 58.5, men: 66.9	89-item FFQ, amount	Upper vs. lower median (reference)	OR	0.77 (0.59, 1.02)	Chungbuk National and Eulji University Hospital	2009 [28]
Dietary iron	Case-control, 1997-1998	136/136, mean age: 57.2, men: 68.4	109-item FFQ, amount	Q4 vs. Q1 (reference)	OR	0.49 (0.24, 1.01)	Hanyang and Hallym University Hospital	2005 [12]
Dietary calcium (1 study)								
Dietary calcium	Case-control, 1997-1998	136/136, mean age: 57.2, men: 68.4	109-item FFQ, amount	Q4 vs. Q1 (reference)	OR	0.43 (0.21, 0.90)	Hanyang and Hallym University Hospital	2005 [12]
Preservation and processing of foods								
Pickled vegetables and kimchi (7 studies)								
Pickled vegetables	Case-control, 2002-2006	307/307, mean age: 56.6, men: 67.1	103/116-item FFQ, amount	T3 vs. T1 (reference)	OR	0.80 (0.52, 1.24)	Chungnam National and Hanyang University Hospital	2021 [29]
Pickled vegetables	Cohort, 1993-2004, mean follow-up: 10.3	81/4,432, mean age: 58.1, men: 38.4	14-item brief FFQ, frequency	Per 40 g/day increment	RR	0.95 (0.80, 1.13)	Korean Multi-Center Cancer Cohort	2020 [30]
Korean cabbage kimchi	Case-control, 2011-2014	415/830, mean age: 53.7, men: 65.1	106-item FFQ, amount	T3 vs. T1 (reference)	OR	1.11 (0.80, 1.55)	National Cancer Center	2016 [15]
Radish kimchi				T3 vs. T1 (reference)		0.80 (0.57, 1.12)		
Chonggak kimchi				T3 vs. T1 (reference)		0.81 (0.58, 1.13)		
Kimchi	Case-control, 2000-2005	471/471, mean age: 58.5, men: 66.9	89-item FFQ, amount	Upper vs. lower median (reference)	OR	3.27 (2.44, 4.37)	Chungbuk National and Eulji University Hospital	2009 [28]
Kimchi	Case-control, 1999	69/199, most frequent age range: 41-55,	161-item FFQ, frequency	≥2 vs. <2/day (reference)	OR	1.90 (1.30, 2.80)	Asan Medical Center	2003 [17]
Kimchi	Case-control, 1997-2001	men: 61.9 214/214, mean age: 58.8, men: 67.5	89-item FFQ, amount	Upper vs. lower median (reference)	OR	1.51 (1.12, 2.44)	Chungbuk National and Eulji University Hospital	2003 [26]
Baiechu kimchi	Case-control, 1997-1998	136/136, mean age: 57.2, men: 68.4	109-item FFQ, amount	Q4 vs. Q1 (reference)	OR	0.50 (0.25, 1.01)	Hanyang and Hallym University Hospital	2002 [18]
Baiechu kimchi stew				Q4 vs. Q1 (reference)		0.62 (0.29, 1.35)		
Kkakduki				Q4 vs. Q1 (reference)		1.78 (0.85, 3.73)		
Dongchimi				Q4 vs. Q1 (reference)		1.96 (1.01, 3.83)		
Salted seafood and fish (4 studies)								
Salted fish	Cohort, 1993-2004, mean follow-up: 12.9	296/11,026 mean age: 57.4, men: 39.1	14-item brief FFQ, frequency	Per 60 g/day increment	RR	1.01 (0.63, 1.61)	Korean Multi-Center Cancer Cohort	2020 [30]
Salt-fermented fish	Case-control, 1999	69/199, most frequent age range: 41-55, men: 61.9	161-item FFQ, frequency	≥1 vs. <1/mo (reference)	OR	2.40 (1.00, 5.70)	Asan Medical Center	2003 [17]
Salted seafood	Case-control, 1997-2001	214/214, mean age: 58.8, men: 67.5	89-item FFQ, amount	Upper vs. lower median (reference)	OR	0.67 (0.45, 1.00)	Chungbuk National and Eulji University Hospital	2003 [26]
Salted fish and shellfish	Case-control, 1997-1998	136/136, mean age: 57.2, men: 68.4	109-item FFQ, amount	Q4 vs. Q1 (reference)	OR	0.78 (0.39, 1.56)	Hanyang and Hallym University Hospital	2002 [18]
Fermented soy products (4 studies)								
Fermented soy paste	Case-control, 2011-2014	377/754, mean age: 53.8, men: 65.3	106-item FFQ, amount	T3 vs. T1 (reference)	OR	1.08 (0.77, 1.51)	National Cancer Center	2017 [23]
Soybean paste	Cohort, 1993-2004, mean follow-up: 8.5	166/9,558, mean age: 57.6, men: 68.4	14-item brief FFQ, frequency	≥1 time/day vs. almost never (reference)	RR	2.01 (0.52, 8.50)	Korean Multi-Center Cancer Cohort	2013 [14]
Soybean paste	Case-control, 2000-2005	471/471, mean age: 58.5, men: 66.9	89-item FFQ, amount	Upper vs. lower median (reference)	OR	1.63 (1.24, 2.14)	Chungbuk National and Eulji University Hospital	2009 [28]
Soybean paste stew	Case-control, 1997-1999	204/204, mean age: 59.5; men: 68.8	84-item FFQ, frequency	≥1/wk vs. <1/mo (reference)	OR	2.73 (1.34, 5.56)	Chungbuk National University Hospital	2000 [19]
Sodium (3 studies)								
Sodium	Cohort, 2004-2008, median follow-up: 7.0	46/7,637, mean age: 48.4, men: 54.8	3-day DR, amount	≥4,000 vs. <4,000 mg/day (reference)	HR	2.34 (1.05, 5.19)	National Cancer Center	2014 [13]
Sodium	Case-control, 2000-2005	471/471, mean age: 58.5, men: 66.9	89-item FFQ, amount	Upper vs. lower median (reference)	OR	2.30 (1.61, 3.30)	Chungbuk National and Eulji University Hospital	2009 [28]
Sodium	Case-control, 1997-1998	136/136, mean age: 57.2, men: 68.4	109-item FFQ, amount	Q4 vs. Q1 (reference)	OR	0.56 (0.28, 1.11)	Hanyang and Hallym University Hospital	2005 [12]
Non-alcoholic drinks								
Coffee (2 studies)								
Coffee	Cross-sectional, 2004-2016	976/161,244, mean age: 53.2, men: 34.3	106-item FFQ, frequency	>60 cups/mo vs. no drink (reference)	OR	0.80 (0.65, 0.98)	KoGES-HEXA	2021 [31]
Coffee	Cohort, 1993-2004, mean follow-up: 8.5	166/9,558, mean age: 57.6, men: 68.4	14-item brief FFQ, frequency	≥1 time/day vs. almost never (reference)	RR	0.94 (0.63, 1.41)	Korean Multi-Center Cancer Cohort	2013 [14]
Tea (2 studies)								
Citrus tea	Case-control, 2011-2014	415/830, mean age: 53.7, men: 65.1	106-item FFQ, amount	T3 vs. T1 (reference)	OR	0.83 (0.59, 1.18)	National Cancer Center	2016 [15]
Tea	Case-control, 1997-1999	204/204, mean age: 59.5, men: 68.8	84-item FFQ, frequency	≥1/wk vs. <1/mo (reference)	OR	0.32 (0.06, 1.61)	Chungbuk National University Hospital	2000 [19]
Other dietary exposures								
Dietary pattern (3 studies)								
Factor analysis: Westernized	Case-control, 2011-2014	415/830, mean age: 53.7, men: 65.1	106-item FFQ, amount	T3 vs. T1 (reference)	OR	0.76 (0.50, 1.16)	National Cancer Center	2021 [32]
Prudent				T3 vs. T1 (reference)		0.58 (0.41, 0.84)		
Index-based: hydrophilic ORAC	Case-control, 2011-2014	415/830, mean age: 53.7, men: 65.1	106-item FFQ, amount	T3 vs. T1 (reference)	OR	0.57 (0.39, 0.82)	National Cancer Center	2020 [33]
Lipophilic ORAC				T3 vs. T1 (reference)		0.66 (0.45, 0.95)		
Total phenolics						0.57 (0.39, 0.83)		
Index-based: DII	Case-control, 2011-2014	388/776, mean age: 53.3, men: 64.2	106-item FFQ, amount	T3 vs. T1 (reference)	OR	1.63 (1.15, 2.29)	National Cancer Center	2017 [34]
Glycemic load (1 study)								
Glycemic index	Case-control, 2002-2006	307/307, mean age: 56.6, men: 67.1	102, 115-item FFQ, amount	T3 vs. T1 (reference)	OR	1.88 (1.18, 2.97)	Hanyang and Chungnam National University Hospital	2022 [35]
Glycemic load				T3 vs. T1 (reference)		2.51 (1.53, 4.12)		
Saturated fat (1 study)								
Saturated fat	Case-control, 1997-1998	136/136, mean age: 57.2, men: 68.4	109-item FFQ, amount	Q4 vs. Q1 (reference)	OR	0.75 (0.37, 1.53)	Hanyang and Hallym University Hospital	2005 [12]
Dietary retinol (1 study)								
Dietary retinol	Case-control, 1997-1998	136/136, mean age: 57.2, men: 68.4	109-item FFQ, amount	Q4 vs. Q1 (reference)	OR	0.57 (0.26, 1.23)	Hanyang and Hallym University Hospital	2005 [12]

OR, odds ratio; RR, relative risk; HR, hazard ratio; CI, confidence interval; Ref, reference number; FFQ, food frequency questionnaire; DR, dietary record; ORAC, oxygen radical absorbance capacity; DII, dietary inflammatory index; KoGES-HEXA, Korean Genome and Epidemiology Study-Health Examinee.

**Table 2.** Literature review on diet and colorectal cancer in Korea

Dietary factors	Study design, enrollment year, follow-up duration (yr)	Sample size (cases/controls, non-cases), age (yr), % of men	Diet assessment, amount or frequency	Risk estimate			Sources	Year [Ref]
				Category	Type	Effect (95% CI)		
Whole grains, fruits, and vegetables								
Dietary fiber (2 studies)								
Dietary fiber	Case-control, 2010-2011	150/116, most frequent age range: 60-69, men: 62.0	102-item FFQ, amount	T3 vs. T1 (reference)	OR	0.22 (0.08, 0.56)	Gangnam Severance Hospital	2015 [36]
Dietary fiber	Case-control	136/134, mean age: 53.3, men: 62.5	93-item FFQ, amount	T3 vs. T1 (reference)	OR	0.20 (0.08, 0.51)	Three university-affiliated hospitals in Seoul (not specified)	2005 [37]
Fruits and vegetables (6 studies)								
Total fruit and vegetables	Case-control, 2007-2014	923/1,846, mean age: 56.3, men: 67.7	106-item FFQ, amount	T3 vs. T1 (reference)	OR	0.60 (0.45, 0.79)	National Cancer Center	2017 [38]
Total fruit						0.77 (0.58, 1.02)		
Total vegetables						0.48 (0.36, 0.64)		
Fruits	Case-control, 2010-2011	150/116, most frequent age range: 60-69, men: 62.0	102-item FFQ, amount	T3 vs. T1(reference)	OR	0.62 (0.27, 1.42)	Gangnam Severance Hospital	2015 [36]
Vegetables						0.54 (0.23, 1.28)		
Fruits and vegetables	Cohort, 2004-2008, median follow-up: 7.0	53/7,637, mean age: 48.4, men: 54.7	3-day DR, amount	≥600 vs. <600 g/day (reference)	HR	0.85 (0.38, 1.92)	National Cancer Center	2014 [13]
Fruits	Case-control	136/134, mean age: 53.3, men: 62.5	93-item FFQ, amount	T3 vs. T1 (reference)	OR	0.38 (0.20, 0.74)	Three university-affiliated hospitals in Seoul (not specified)	2005 [37]
Vegetables						0.30 (0.15, 0.62)		
Fruits 1	Case-control, 1994-1999	(Men) 86/899, mean age: 46.3	51-item FFQ, amount	Q4 vs. Q1 (reference)	OR	0.53 (0.22, 1.27)	Our Lady of Mercy Hospital (Catholic University)	2005 [39]
Fruits 2						0.36 (0.16, 0.84)		
Green/yellow vegetables 1 (fresh)						0.97 (0.40, 2.35)		
Green/yellow vegetables 2 (fresh)						1.33 (0.39, 4.52)		
Green/yellow vegetables 1 (boiling)						0.75 (0.33, 1.71)		
Green/yellow vegetables 2 (boiling)						0.92 (0.38, 2.23)		
Light color vegetables 1 (fresh)						0.64 (0.19, 2.10)		
Light color vegetables 2 (fresh)						0.65 (0.19, 2.16)		
Light color vegetables 1 (boiling)						0.84 (0.33, 2.18)		
Light color vegetables 2 (boiling)						0.45 (0.15, 1.39)		
Fruits 1	Case-control, 1994-1999	(Women) 76/1,677, mean age: 47.2	51-item FFQ, amount	Q4 vs. Q1 (reference)	OR	1.13 (0.49, 2.61)	Our Lady of Mercy Hospital (Catholic University)	2005 [39]
Fruits 2						1.14 (0.54, 2.40)		
Green/yellow vegetables 1 (fresh)						0.45 (0.15, 1.36)		
Green/yellow vegetables 2 (fresh)						0.89 (0.31, 2.57)		
Green/yellow vegetables 1 (boiling)						0.80 (0.30, 2.11)		
Green/yellow vegetables 2 (boiling)						1.17 (0.49, 2.81)		
Light color vegetables 1 (fresh)						0.52 (0.11, 2.35)		
Light color vegetables 2 (fresh)						0.97 (0.28, 3.35)		
Light color vegetables 1 (boiling)						0.46 (0.18, 1.16)		
Light color vegetables 2 (boiling)						0.71 (0.27, 1.83)		
Vegetables	Case-control	125/247, mean age: 56.5, men: 63.0	Not specified, frequency	High vs. low (reference)	OR	0.80 (0.49, 1.31)	Ilsan-Paik Hospital	2003 [40]
Dietary carotenoids (3 studies)								
Dietary lutein/zeaxanthin	Case-control, 2007-2014	923/1,846, mean age: 56.3, men: 67.7	106-item FFQ, amount	Q4 vs. Q1(reference)	OR	0.25 (0.18, 0.36)	National Cancer Center	2019 [41]
Dietary β-carotene	Case-control, 2010-2011	150/116, most frequent age range: 60-69, men: 62.0	102-item FFQ, amount	T3 vs. T1 (reference)	OR	0.56 (0.17, 1.87)	Gangnam Severance Hospital	2015 [36]
Dietary carotene	Case-control	136/134, mean age: 53.3, men: 62.5	93-item FFQ, amount	T3 vs. T1 (reference)	OR	0.12 (0.06, 0.28)	Three university-affiliated hospitals in Seoul (not specified)	2005 [37]
Dietary vitamin C (2 studies)								
Dietary vitamin C	Case-control, 2010-2011	150/116, most frequent age range: 60-69, men: 62.0	102-item FFQ, amount	T3 vs. T1 (reference)	OR	0.38 (0.14, 1.05)	Gangnam Severance Hospital	2015 [36]
Dietary vitamin C	Case-control	136/134, mean age: 53.3, men: 62.5	93-item FFQ, amount	T3 vs. T1 ((reference)	OR	0.18 (0.08, 0.40)	Three university-affiliated hospitals in Seoul (not specified)	2005 [37]
Dietary Isoflavone (1 study)								
Dietary isoflavone	Case-control, 2007-2014	923/1,846, mean age: 56.3, men: 67.7	106-item FFQ, amount	Q4 vs. Q1 (reference)	OR	0.61 (0.46, 0.81)	National Cancer Center	2017 [42]
Meat, fish, and dairy products								
Meat (8 studies)								
Meat	Cohort, 1996-1997, follow-up: 6.0-7.0	6444/2,241,685, most frequent age range: 40-49, men: 36.8	A single question, frequency	≥4 vs. ≤1/wk (reference)	HR	1.23 (1.13, 1.35)	Korean Health Insurance Corporation	2011 [43]
Meat	Case-control, 2003-2005	80/75, mean age: 57.1, men: 52.0	A single question, frequency	≥3/wk vs. none (reference)	OR	1.7 (0.70, 4.20)	Ewha Womans University Hospital	2006 [44]
Meat	Case-control	125/247, mean age: 56.5, men: 63.0	Not specified, frequency	>2 vs. <2/wk (reference)	OR	1.72 (1.12, 2.76)	Ilsan-Paik Hospital	2003 [40]
Red meat	Case-control, 2007-2014	703/1,406, mean age: 56.1, men: 68.3	106-item FFQ, amount	≥100 vs. <100 g/day (reference)	OR	0.66 (0.47, 0.92)	National Cancer Center	2019 [45]
Processed meat				≥50 vs. <50 g/day (reference)		0.78 (0.16, 3.93)		
Red meat	Case-control, 1995-2004	971/658, mean age: 58.2, men: 56.2	94-item FFQ, frequency	≥5 vs. <1/wk (reference)	OR	1.29 (0.83, 2.01)	Three university-affiliated hospitals in Seoul (not specified)	2019 [46]
Red meat	Case-control, 2010-2011	150/116, most frequent age range: 60-69, men: 62.0	102-item FFQ, amount	T3 vs. T1 (reference)	OR	7.33 (2.98, 18.06)	Gangnam Severance Hospital	2015 [36]
Red meat	Cohort, 2004-2008, median follow-up: 7.0	53/7,637, mean age: 48.4, men: 54.7	3-day DR, amount	≥600 vs. <600 g/day (reference)	HR	1.31 (0.60, 2.61)	National Cancer Center	2014 [13]
Beef	Case-control	136/134, mean age: 53.3, men: 62.5	93-item FFQ, amount	T3 vs. T1 (reference) T3 vs. T1 (reference)	OR	0.62 (0.30, 1.28)	Three university-affiliated hospitals in Seoul (not specified)	2005 [37]
Pork						1.70 (0.80, 3.58)		
Fish (2 studies)								
Fish	Case-control, 2010-2011	150/116, most frequent age range: 60-69, men: 62.0	102-item FFQ, amount	T3 vs. T1 (reference)	OR	1.05 (0.45, 2.40)	Gangnam Severance Hospital	2015 [36]
Fish	Case-control	136/134, mean age: 53.3, men: 62.5	93-item FFQ, amount	T3 vs. T1(reference)	OR	2.01 (0.97, 4.18)	Three university-affiliated hospitals in Seoul (not specified)	2005 [37]
Anchovy						0.35 (0.17, 0.74)		
Dairy products (3 studies)								
Dairy	Case-control, 2007-2014	703/1,406, mean age: 56.1, men: 68.3	106-item FFQ, amount	≥400 vs. <400 g/day (reference)	OR	2.23 (1.53, 3.25)	National Cancer Center	2019 [45]
Milk and dairy product	Case-control, 2010-2011	150/116, most frequent age range: 60-69, men: 62.0	102-item FFQ, amount	T3 vs. T1 (reference) OR 2.42 (1.10, 5.31)			Gangnam Severance Hospital	2015 [36]
Milk	Case-control	136/134, mean age: 53.3, men: 62.5	93-item FFQ, amount	T3 vs. T1 (reference)		0.33 (0.18, 0.64)	Three university-affiliated hospitals in Seoul (not specified)	2005 [37]
Dietary iron (1 study)								
Dietary iron	Case-control	136/134, mean age: 53.3, men: 62.5	93-item FFQ, amount	T3 vs. T1 (reference)	OR	0.49 (0.18, 1.30)	Three university-affiliated hospitals in Seoul (not specified)	2005 [37]
Dietary calcium (3 studies)								
Dietary calcium	Cohort, 2004-2013 mean follow-up: 5.4	635/118,866, mean age: 52.7, men: 34.3	106-item FFQ, amount	Per 200 g/day	HR	0.93 (0.86, 1.01)	KoGES-HEXA	2021 [47]
Dietary calcium	Case-control, 2007-2014	(Men) 624/1,872, most frequent age range: 50-59	106-item FFQ, amount	Q4 vs. Q1 (reference)	OR	0.16 (0.11, 0.24)	National Cancer Center	2015 [48]
Dietary calcium	Case-control, 2007-2014	(Women) 298/894, most frequent age range: 50-59	106-item FFQ, amount	Q4 vs. Q1 (reference)	OR	0.16 (0.09, 0.29)	National Cancer Center	2015 [48]
Dietary calcium	Case-control	136/134, mean age: 53.3, men: 62.5	93-item FFQ, amount	T3 vs. T1 (reference)	OR	0.18 (0.07, 0.42)	Three university-affiliated hospitals in Seoul (not specified)	2005 [37]
Preservation and processing of foods								
Kimchi (2 studies)								
Kimchi	Case-control	136/134, mean age: 53.3, men: 62.5	93-item FFQ, amount	T3 vs. T1 (reference)	OR	0.32 (0.15, 0.65)	Three university-affiliated hospitals in Seoul (not specified)	2005 [37]
Kimchi	Case-control, 1994-1999	(Men) 86/899, mean age: 46.3	51-item FFQ, amount	Q4 vs. Q1 (reference)	OR	1.31 (0.72, 2.38)	Our Lady of Mercy Hospital (Catholic University)	2005 [39]
Kimchi	Case-control, 1994-1999	(Women) 76/1,677, mean age: 47.2	51-item FFQ, amount	Q4 vs. Q1 (reference)	OR	0.99 (0.59, 1.68)	Our Lady of Mercy Hospital (Catholic University)	2005 [39]
Fermented soy products (1 study)								
Fermented soy paste	Case-control, 2007-2014	(Men) 624/1,872, most frequent age range: 50-59	106-item FFQ, amount	Q4 vs. Q1 (reference)	OR	1.82 (1.35, 2.46)	National Cancer Center	2015 [49]
		(Women) 298/894, most frequent age range: 50-59	106-item FFQ, amount	Q4 vs. Q1 (reference)	OR	1.22 (0.77, 1.91)	National Cancer Center	2015 [49]
Sodium (2 studies)								
Sodium	Case-control, 2010-2011	150/116, most frequent age range: 60-69, men: 62.0	102-item FFQ, amount	T3 vs. T1 (reference)	OR	0.95 (0.39, 2.32)	Gangnam Severance Hospital	2015 [36]
Sodium	Cohort, 2004-2008, median follow-up: 7.0	53/7,637, mean age: 48.4, men: 54.7	3-day DR, amount	≥4,000 vs. <4,000 mg/day (reference)	HR	1.52 (0.75, 3.08)	National Cancer Center	2014 [13]
Non-alcoholic drinks								
Coffee (2 studies)								
Coffee	Case-control, 2007-2014	923/1,846, mean age: 56.3, men: 67.7	106-item FFQ, frequency	≥3 cups/day vs. none (reference)	OR	0.22 (0.14, 0.33)	National Cancer Center	2021 [50]
Coffee	Cross-sectional, 2004-2016	521/161,699, mean age: 53.2, men: 34.3	106-item FFQ, frequency	>60 cups/mo vs. no drink (reference)	OR	0.53 (0.39, 0.72)	KoGES-HEXA	2021 [31]
Tea (1 study)								
Green tea	Case-control, 2007-2014	922/1,820, mean age: 56.3, men: 67.8	106-item FFQ, amount	T3 vs. T1 (reference)	OR	0.59 (0.46, 0.76)	National Cancer Center	2019 [51]
Other dietary exposures								
Dietary pattern (5 studies)								
Index-based: DIS	Case-control, 2007-2014	919/1,846, mean age: 56.3, men: 67.7	106-item FFQ, amount	T3 vs. T1 (reference)	OR	3.00 (2.19, 4.10)	National Cancer Center	2022 [52]
Index-based: EDIH	Case-control, 2007-2014	923/1,846, mean age: 56.3, men: 67.7	106-item FFQ, amount	Q4 vs. Q1 (reference)	OR	1.14 (0.81, 1.60)	National Cancer Center	2022 [53]
EDIR						3.32 (2.32, 4.74)		
RRR: CRP-related pattern	Case-control, 2007-2014	695/1,846, mean age: 56.2, men: 67.8	106-item FFQ, amount	Q4 vs. Q1 (reference)	OR	9.98 (6.81, 14.62)	National Cancer Center	2018 [54]
Factor analysis: traditional diet	Case-control, 2007-2014	923/1,846, mean age: 56.3, men: 67.7	106-item FFQ, amount	T3 vs. T1 (reference)	OR	0.35 (0.27, 0.46)	National Cancer Center	2016 [55]
Westernized diet				T3 vs. T1 (reference)		2.35 (1.78, 3.09)		
Prudent diet				T3 vs. T1 (reference)		0.37 (0.28, 0.48)		
Index-based: DII	Case-control, 2007-2014	923/1,846, mean age: 56.3, men: 67.7	106-item FFQ, amount	T3 vs. T1 (reference)	OR	2.16 (1.71, 2.73)	National Cancer Center	2016 [56]
Glycemic load (1 study)								
Glycemic index	Case-control, 2007-2014	695/1,401, mean age: 56.1, men: 68.3	106-item FFQ, amount	T3 vs. T1 (reference)	OR	5.44 (3.85, 7.68)	National Cancer Center	2022 [57]
Glycemic load				T3 vs. T1 (reference)		4.43 (3.18, 6.15)		
Saturated fat (2 studies)								
Saturated fatty acids	Case-control, 2010-2011	150/116, most frequent age range: 60-69, men: 62.0	102-item FFQ, amount	T3 vs. T1 (reference)	OR	2.96 (1.24, 7.04)	Gangnam Severance Hospital	2015 [36]
Saturated fatty acids	Case-control	136/134, mean age: 53.3, men: 62.5	93-item FFQ, amount	T3 vs. T1 (reference)	OR	0.46 (0.21, 0.99)	Three university-affiliated hospitals in Seoul (not specified)	2005 [37]
Dietary retinol (1 study)								
Dietary retinol	Case-control	136/134, mean age: 53.3, men: 62.5	93-item FFQ, amount	T3 vs. T1 (reference)	OR	0.65 (0.31, 1.35)	Three university-affiliated hospitals in Seoul (not specified)	2005 [37]
Dietary vitamin D (1 study)								
Dietary vitamin D	Case-control, 2010-2011	150/116, most frequent age range: 60-69, men: 62.0	102-item FFQ, amount	T3 vs. T1 (reference)	OR	0.79 (0.37, 1.67)	Gangnam Severance Hospital	2015 [36]

OR, odds ratio; RR, relative risk; HR, hazard ratio; CI, confidence interval; Ref, reference number; FFQ, food frequency questionnaire; DR, dietary record; DIS, dietary inflammation score; EDIH, empirical dietary index for hyperinsulinemia; EDIR, empirical dietary index for insulin resistance; RRR, reduced rank regression; DII, dietary inflammatory index; KoGES-HEXA, Korean Genome and Epidemiology Study-Health Examinee.

**Table 3.** Literature review on diet and breast cancer in Korea

Dietary factors	Study design, enrollment year, follow-up duration (yr)	Sample size (cases/controls, non-cases), age (yr), % of women	Diet assessment, amount or frequency	Risk estimate			Sources	Year [Ref]
				Category	Type	Effect (95% CI)		
Whole grains, fruits, and vegetables								
Dietary fiber (2 studies)								
Dietary fiber	Case-control, 2004-2005	103/159, mean age: 50.1, women: 100	74-item FFQ, amount	Q4 vs. Q1 (reference)	OR	0.37 (0.14, 0.99)	Daegu-area hospital for cases and community controls	2008 [58]
Dietary fiber	Case-control, 1998-1999	108/121, most frequent age range: 40-49, women: 100	98-item FFQ, amount	Q4 vs. Q1 (reference)	OR	0.61 (0.31, 2.06)	Hanyang and Soonchunhyang University Hospitals	2000 [59]
Fruits and vegetables (5 studies)								
Fruits	Cohort, 2002-2007, mean follow-up: 9.5	72/4,974, most frequent age range: 40-49, women: 100	16-item brief FFQ, frequency	≥1/day vs. ≤4-6/wk (reference)	HR	1.22 (0.76, 1.97)	National Cancer Center	2017 [60]
Light-colored vegetables				≥4-6 vs. ≤2-3/wk (reference)		0.87 (0.54, 1.38)		
Green-yellow vegetables				≥1/day vs. ≤4-6/wk (reference)		1.46 (0.91, 2.33)		
Total fruit and vegetables	Case-control, 2007-2008	358/360, mean age: 48.1, women: 100	103-item FFQ, amount	Q4 vs. Q1 (reference)	OR	0.34 (0.19, 0.62)	National Cancer Center	2010 [61]
Fruits				Q4 vs. Q1 (reference)		0.75 (0.44, 1.28)		
Total vegetables				Q4 vs. Q1 (reference)		0.22 (0.12, 0.41)		
Non-pickled vegetables				Q4 vs. Q1 (reference)		0.09 (0.05, 0.18)		
Total fruit	Case-control, 1999-2003	359/708, mean age: 49.1, women: 100	98-item FFQ, amount	Q4 vs. Q1 (reference)	OR	0.79 (0.52, 1.32)	Hanyang and Soonchunhyang University Hospitals	2007 [62]
Citrus fruit				Q4 vs. Q1 (reference)		0.74 (0.40, 1.28)		
Total vegetables				Q4 vs. Q1 (reference)		0.76 (0.46, 1.23)		
Fruits	Case-control, 2004-2005	103/159, mean age: 50.1, women: 100	22-item FFQ, frequency	1/day vs. ≤1/wk (reference)	OR	0.37 (0.15, 0.90)	Daegu-area hospital for cases and community controls	2007 [63]
Green-yellow color vegetables				1/day vs. ≤1/wk (reference)		0.83 (0.26, 2.68)		
Light color vegetables				1/day vs. ≤1/wk (reference)		0.58 (0.22, 1.53)		
Fruits	Case-control, 1995-2002	819/713, mean age: 47.4, women: 100	FFQ, frequency	Everyday vs. <1/day (reference)	OR	0.70 (0.60, 0.90)	Seoul National University Hospital, Asan Medical Center, and Seoul Metropolitan Government Seoul National University Boramae Medical Center	2003 [64]
Green vegetables				Everyday vs. <1/day (reference)		0.60 (0.40, 1.00)		
White vegetables				Everyday vs. <1/day (reference)		1.10 (0.80, 1.50)		
Dietary carotenoids (4 studies)								
Dietary β-carotene	Case-control, 2001-2003	512/512, mean age: 48.8, women: 100	56-item FFQ, amount	Q4 vs. Q1 (reference)	OR	0.80 (0.53, 1.20)	Seoul National University Hospital, Asan Medical Center, and Ewha Womans University Hospital	2012 [65]
Dietary β-carotene	Case-control, 2004-2006	362/362, mean age: 46.1, women: 100	121-item FFQ, amount	Per 500 ug/day	OR	1.01 (0.98, 1.05)	Samsung Medical Center	2010 [66]
Dietary β-carotene	Case-control, 2004-2005	103/159, mean age: 50.1, women: 100	74-item FFQ, amount	Q4 vs. Q1 (reference)	OR	0.80 (0.33. 1.95)	Daegu-area hospital for cases and community controls	2008 [58]
Dietary β-carotene	Case-control, 1999-2000	224/250, most frequent age range: 40-59, women: 100	98-item FFQ, amount	Q4 vs. Q1 (reference)	OR	0.42 (0.25, 0.89)	Hanyang and Soonchunhyang University Hospitals	2003 [67]
Dietary vitamin C (5 studies)								
Dietary vitamin C	Cohort, 2004-2013, mean follow-up: 4.9	232/40,200, most frequent age range: 40-59, women: 100	103-item FFQ, amount	>100 vs. ≤100 mg/day (reference)	HR	0.95 (0.71, 1.26)	KoGES-HEXA	2022 [68]
Dietary vitamin C	Case-control, 2001-2003	512/512, mean age: 48.8, women: 100	56-item FFQ, amount	Q4 vs. Q1 (reference)	OR	1.07 (0.72, 1.60)	Seoul National University Hospital, Asan Medical Center, and Ewha Womans University Hospital	2012 [65]
Dietary vitamin C	Case-control, 2004-2006	362/362, mean age: 46.1, women: 100	121-item FFQ, amount	Per 10 mg/day	OR	1.01 (0.99, 1.04)	Samsung Medical Center	2010 [66]
Dietary vitamin C	Case-control, 2004-2005	103/159, mean age: 50, women: 100	74-item FFQ, amount	Q4 vs. Q1 (reference)	OR	0.76 (0.30, 1.93)	Daegu-area hospital for cases and community controls	2008 [58]
Dietary vitamin C	Case-control, 1999-2000	224/250, most frequent age range: 40-59, women: 100	98-item FFQ, amount	Q4 vs. Q1(reference)	OR	0.37 (0.19, 0.84)	Hanyang and Soonchunhyang University Hospitals	2003 [67]
Dietary isoflavone (1 study)								
Dietary isoflavone	Case-control, 2007-2008	358/360, mean age: 48.1, women: 100	103-item FFQ, amount	Q4 vs. Q1 (reference)	OR	0.81 (0.48, 1.38)	National Cancer Center	2010 [69]
Meat, fish, and dairy products								
Meat (4 studies)								
Low fat meat	Case-control, 2004-2005	103/159, mean age: 50.1, women: 100	22-item FFQ, frequency	2-3 vs. ≤1/wk (reference)	OR	0.64 (0.38, 1.09)	Daegu-area hospital for cases and community controls	2007 [63]
High fat meat				2-3 vs. ≤1/wk (reference)		0.79 (0.40, 1.53)		
Meat	Case-control, 1995-2002	819/713, mean age: 47.4, women: 100	FFQ, frequency	≥1 vs. <1/wk (reference)	OR	1.50 (1.20, 1.90)	Seoul National University Hospital, Asan Medical Center, and Seoul Metropolitan Government Seoul National University Boramae Medical Center	2003 [64]
Grilled meat	Cohort, 2002-2007, mean follow-up: 9.5	72/4,974, most frequent age range: 40-49, women: 100	16-item brief FFQ, frequency	≥2-3 vs. ≤1/mo (reference)	HR	1.77 (1.09, 2.85)	National Cancer Center	2017 [60]
Grill beef rib	Case-control, 1998-1999	108/121, most frequent age range: 40-49, women: 100	98-item FFQ, amount	Q4 vs. Q1 (reference)	OR	0.96 (0.63, 2.02)	Hanyang and Soonchunhyang University Hospitals	2000 [59]
Bulgogi						1.12 (0.73, 2.38)		
Grilled pork						1.21 (0.89, 2.21)		
Grilled pork belly						1.11 (0.81, 2.15)		
Pork cutlet						0.91 (0.78, 2.61)		
Grilled ham						0.87 (0.71, 2.18)		
Fish (5 studies)								
Bony fish	Cohort, 2002-2007, mean follow-up: 9.5	72/4,974, most frequent age range: 40-49, women: 100	16-item brief FFQ, frequency	≥2-3 vs. ≤1/wk (reference)	HR	1.14 (0.71, 1.83)	National Cancer Center	2017 [60]
Total fish	Case-control, 2007-2008	358/360, mean age: 48.1, women: 100	103-item FFQ, amount	Q4 vs. Q1 (reference)	OR	0.55 (0.32, 0.96)	National Cancer Center	2009 [70]
Lean fish						1.21 (0.72, 2.04)		
Fatty fish						0.23 (0.13, 0.42)		
White flesh fish	Case-control, 2004-2005	103/159, mean age: 50.1, women: 100	22-item FFQ, frequency	1/day vs. ≤1/wk (reference)	OR	1.64 (0.52–5.16)	Daegu-area hospital for cases and community controls	2007 [63]
Blue flesh fish				≥2-3 vs. ≤1/wk (reference)		1.32 (0.74, 2.36)		
Fish	Case-control, 1995-2002	819/713, mean age: 47.4, women: 100	FFQ, frequency	≥1 vs. <1/wk (reference)	OR	1.50 (1.20, 1.90)	Seoul National University Hospital, Asan Medical Center, and Seoul Metropolitan Government Seoul National University Boramae Medical Center	2003 [64]
Fish meat	Case-control, 1998-1999	108/121, most frequent age range: 40-49, women: 100	98-item FFQ, amount	Q4 vs. Q1 (reference)	OR	0.95 (0.87, 2.44)	Hanyang and Soonchunhyang University Hospitals	2000 [59]
Raw croaker						0.51 (0.35, 1.19)		
Grilled yellow croaker						0.89 (0.21, 1.93)		
Tuna canned						0.85 (0.39, 1.39)		
Dairy products (5 studies)								
Milk	Cohort, 2004-2013, mean follow-up: 6.3	359/77,961, mean age: 52.3, women: 100	106-item FFQ, frequency	≥1/day vs. <1/wk (reference)	HR	0.78 (0.59, 1.04)	KoGES-HEXA Gem	2020 [71]
Dairy food	Cohort, 2002-2007, mean follow-up: 9.5	72/4,974, most frequent age range: 40-49, women: 100	16-item brief FFQ, frequency	≥4-6 vs. ≤2-3/wk (reference)	HR	1.32 (0.83, 2.11)	National Cancer Center	2017 [60]
Milk, yogurt	Case-control, 2004-2005	103/159, mean age: 50.1, women: 100	22-item FFQ, frequency	1/day vs. ≤1/wk (reference)	OR	1.19 (0.52, 2.70)	Daegu-area hospital for cases and community controls	2007 [63]
Milk	Case-control, 1995-2002	819/713, mean age: 47.4, women: 100	FFQ, frequency	Everyday vs. <1/day (reference)	OR	0.90 (0.80, 1.20)	Seoul University Hospital, Asan Medical Center, and Seoul Metropolitan Government Seoul National University Boramae Medical Center	2003 [64]
Milk	Case-control, 1998-1999	108/121, most frequent age range: 40-49, women: 100	98-item FFQ, amount	Q4 vs. Q1 (reference)	OR	0.51 (0.34, 2.20)	Hanyang and Soonchunhyang University Hospitals	2000 [59]
Yogurt						1.05 (0.39, 2.19)		
Cheese						0.51 (0.43, 2.23)		
Dietary iron (3 studies)								
Dietary iron	Cohort, 2004-2013, mean follow-up: 4.9	232/40,200, most frequent age range: 40-59, women: 100	103-item FFQ, amount	>14 vs. ≤14 mg/day (reference) for 30-49 yr, >8 vs. ≤8 mg/day (reference) for 50-74 yr, and >7 vs. ≤7 mg/day (reference) for ≥75 yr	HR	0.74 (0.52, 1.06)	KoGES-HEXA	2022 [68]
Dietary iron	Case-control, 2004-2005	103/159, mean age: 50.1, women: 100	74-item FFQ, amount	Q4 vs. Q1(reference)	OR	0.76 (0.27, 2.16)	Daegu-area hospital for cases and community controls	2008 [58]
Dietary iron	Case-control, 1998-1999	108/121, most frequent age range: 40-49, women: 100	98-item FFQ, amount	Q4 vs. Q1 (reference)	OR	0.71 (0.53, 1.72)	Hanyang and Soonchunhyang University Hospitals	2000 [59]
Dietary calcium (3 studies)								
Dietary calcium	Cohort, 2004-2013, mean follow-up: 4.9	232/40,200, most frequent age range: 40-59, women: 100	103-item FFQ, amount	>700 vs. ≤700 mg/day (reference) for 30-49 yr, >800 vs. ≤800 mg/day (reference) for ≥50 yr	HR	1.12 (0.72, 1.76)	KoGES-HEXA	2022 [68]
Dietary calcium	Case-control, 2004-2005	103/159, mean age: 50.1, women: 100	74-item FFQ, amount	Q4 vs. Q1 (reference)	OR	0.33 (0.13, 0.86)	Daegu-area hospital for cases and community controls	2008 [58]
Dietary calcium	Case-control, 1998-1999	108/121, most frequent age range: 40-49, women: 100	98-item FFQ, amount	Q4 vs. Q1 (reference)	OR	0.85 (0.27, 1.30)	Hanyang and Soonchunhyang University Hospitals	2000 [59]
Preservation and processing of foods								
Pickled vegetables and Kimchi (2 studies)								
Pickled vegetables	Case-control, 2007-2008	358/360, mean age: 48.1, women: 100	103-item FFQ, amount	Q4 vs. Q1 (reference)	OR	2.47 (1.45, 4.21)	National Cancer Center	2010 [61]
Cabbage kimchi	Case-control, 1999-2003	359/708, mean age: 49.1, women: 100	98-item FFQ, amount	Q4 vs. Q1 (reference)	OR	0.83 (0.57, 1.59)	Hanyang and Soonchunhyang University Hospitals	2007 [62]
Radish kimchi						0.77 (0.45, 1.27)		
Salted vegetables and fish (1 study)								
Salted vegetables and seafood	Cohort, 2002-2007, mean follow-up: 9.5	72/4,974, most frequent age range: 40-49, women: 100	16-item brief FFQ, frequency	≥2 vs. ≤1/day (reference)	HR	0.98 (0.61, 1.58)	National Cancer Center	2017 [60]
Fermented soy products (2 studies)								
Fermented soy paste	Case-control, 2007-2008	358/360, mean age: 48.1, women: 100	103-item FFQ, amount	Q4 vs. Q1 (reference)	OR	0.31 (0.17, 0.56)	National Cancer Center Hanyang and	2010 [69]
Soybean paste	Case-control, 1999-2003	359/708, mean age: 49.1, women: 100	98-item FFQ, amount	Q4 vs. Q1 (reference)	OR	0.71 (0.54, 1.30)	Soonchunhyang University Hospitals	2007 [62]
Sodium (1 study)								
Sodium	Case-control, 1998-1999	108/121, most frequent age range: 40-49, women: 100	98-item FFQ, amount	Q4 vs. Q1 (reference)	OR	0.96 (0.57, 1.38)	Hanyang and Soonchunhyang University Hospitals	2000 [59]
Non-alcoholic drinks								
Coffee (3 studies)								
Coffee	Cross-sectional, 2004-2016	1117/105,493, mean age: 53.2, women: 100	106-item FFQ, frequency	>60 cups/mo vs. no drink (reference)	OR	0.56 (0.45, 0.70)	KoGES-HEXA	2021 [31]
Coffee	Case-control, 2004-2005	103/159, mean age: 50.1, women: 100	22-item FFQ, frequency	1/day vs. ≤1/wk (reference)	OR	1.17 (0.61, 2.25)	Daegu-area hospital for cases and community controls	2007 [63]
Coffee	Case-control, 1998-1999	108/121, most frequent age range: 40-49, women: 100	98-item FFQ, amount	Q4 vs. Q1 (reference)	OR	0.53 (0.44, 1.23)	Hanyang and Soonchunhyang University Hospitals	2000 [59]
Tea (2 studies)								
Green tea	Case-control, 2004-2005	103/159, mean age: 50.1, women: 100	22-item FFQ, frequency	1/day vs. ≤1/wk (reference)	OR	0.97 (0.49, 1.95)	Daegu-area hospital for cases and community controls	2007 [63]
Green tea	Case-control, 1998-1999	108/121, most frequent age range: 40-49, women: 100	98-item FFQ, amount	Q4 vs. Q1 (reference)	OR	0.58 (0.27, 1.08)	Hanyang and Soonchunhyang University Hospitals	2000 [59]
Other dietary exposures								
Dietary pattern (4 studies)								
Factor analysis: meat diet	Cohort, 2004-2013, mean follow-up: 6.3	359/77,961, mean age: 52.3, women: 100	106-item FFQ, amount	Q4 vs. Q1 (reference)	HR	1.05 (0.76, 1.47)	KoGES-HEXA Gem	2020 [72]
White rice diet				Q4 vs. Q1 (reference)		1.35 (1.00, 1.84)		
Other diet				Q4 vs. Q1 (reference)		1.30 (0.94, 1.80)		
Index-based: DII	Case-control, 2007-2008	364/364, mean age: 47.8, women: 100	106-item FFQ, amount	T3 vs. T1 (reference)	OR	3.68 (2.34, 5.80)	National Cancer Center	2019 [73]
RRR: glycemic index-based pattern, Glycemic load-based pattern	Case-control, 2007-2008	357/357, mean age: 48.2, women: 100	103-item FFQ, amount	T3 vs. T1 (reference)	OR	1.97 (1.14, 3.42)	National Cancer Center	2013 [74]
				T3 vs. T1 (reference)		2.66 (1.57, 4.49)		
Factor analysis: vegetables-seafood	Case-control, 2007-2008	357/357, mean age: 48.2, women: 100	103-item FFQ, amount	T3 vs. T1 (reference)	OR	0.14 (0.08, 0.25)	National Cancer Center	2010 [75]
Meat-Starch				T3 vs. T1 (reference)		0.69 (0.40, 1.16)		
Glycemic load (2 studies)								
Glycemic index	Case-control, 2007-2008	357/357, mean age: 48.2, women: 100	103-item FFQ, amount	T3 vs. T1 (reference)	OR	2.50 (1.46, 4.31)	National Cancer Center	2013 [74]
Glycemic load				T3 vs. T1 (reference)		3.27 (1.94, 5.50)		
Glycemic index	Case-control, 2004-2006	362/362, mean age: 46.1, women: 100	121-item FFQ, amount	Q5 vs. Q1 (reference)	OR	0.44 (0.23, 0.85)	Samsung Medical Center	2010 [76]
Glycemic load				Q5 vs. Q1 (reference)		0.85 (0.48, 1.50)		
Saturated fat (2 studies)								
Saturated fatty acids	Case-control, 2004-2005	103/159, mean age: 50.1, women: 100	74-item FFQ, amount	Q4 vs. Q1 (reference)	OR	0.22 (0.09, 0.56)	Daegu-area hospital for cases and community controls	2008 [58]
Saturated fatty acids	Case-control, 1999-2000	224/250, most frequent age range: 40-59, women: 100	98-item FFQ, amount	Q4 vs. Q1 (reference)	OR	1.65 (0.92, 2.45)	Hanyang and Soonchunhyang University Hospitals	2003 [67]
Dietary retinol (3 studies)								
Dietary retinol	Case-control, 2001-2003	512/512, mean age: 48.8, women: 100	56-item FFQ, amount	Q4 vs. Q1 (reference)	OR	0.72 (0.45, 1.16)	Seoul National University Hospital, Asan Medical Center, and Ewha Womans University Hospital	2012 [65]
Dietary retinol	Case-control, 2004-2005	103/159, mean age: 50.1, women: 100	74-item FFQ, amount	Q4 vs. Q1 (reference)	OR	0.62 (0.23, 1.67)	Daegu-area hospital for cases and community controls	2008 [58]
Dietary retinol	Case-control, 1999-2000	224/250, most frequent age range: 40-59, women: 100	98-item FFQ, amount	Q4 vs. Q1 (reference)	OR	0.88 (0.26, 1.09)	Hanyang and Soonchunhyang University Hospitals	2003 [67]

OR, odds ratio; RR, relative risk; HR, hazard ratio; CI, confidence interval; Ref, reference number; FFQ, food frequency questionnaire; DII, dietary inflammatory index; RRR, reduced rank regression; KoGES-HEXA, Korean Genome and Epidemiology Study-Health Examinee.

**Table 4.** Literature review on diet and thyroid cancer in Korea

Dietary factors	Study design, enrollment year, follow-up duration (yr)	Sample size (cases/controls, non-cases), age (yr), % of men	Diet assessment, amount or frequency	Risk estimate			Sources	Year [Ref[
				Category	Type	Effect (95% CI)		
Whole grains, fruits, and vegetables								
Dietary fiber (1 study)								
Dietary fiber	Case-control, 2007-2014	113/226, mean age: 53.7, men: 0.0	106-item FFQ, amount	Upper vs. lower median (reference)	OR	1.18 (0.75, 1.87)	National Cancer Center	2016 [77]
Fruits and vegetables (2 studies)								
Fruits and vegetables	Cohort, 2004-2008, median follow-up: 7.0	136/7,637, mean age: 48.4, men: 54.6	3-day DR, amount	≥600 vs. <600 g/day (reference)	HR	0.87 (0.54, 1.42)	National Cancer Center	2014 [13]
Total fruit	Case-control, 2008-2010	111/111, mean age: 45.6, men: 0.0	121-item FFQ, amount	Q4 vs. Q1 (reference)	OR	0.59 (0.23, 1.52)	Hanyang University Hospital	2013 [78]
Total vegetables				Q4 vs. Q1 (reference)		0.51 (0.15, 1.78)		
Raw vegetables				Q4 vs. Q1 (reference)		0.20 (0.07, 0.62)		
Carotenoid (1 study)								
Dietary β-carotene	Case-control, 2007-2014	113/226, mean age: 53.7, men: 0.0	106-item FFQ, amount	Upper vs. lower median (reference)	OR	1.22 (0.77, 1.93)	National Cancer Center	2016 [77]
Dietary vitamin C (1 study)								
Dietary vitamin C	Case-control, 2007-2014	113/226, mean age: 53.7, men: 0.0	106-item FFQ, amount	Upper vs. lower median (reference)	OR	1.17 (0.74, 1.85)	National Cancer Center	2016 [77]
Meat, fish, and dairy products								
Red meat (1 study)								
Red meat	Cohort, 2004-2008, median follow-up: 7.0	136/7,637, mean age: 48.4, men: 54.6	3-day DR, amount	≥43 vs. <43 g/day (reference)	HR	0.91 (0.61, 1.36)	National Cancer Center	2014 [13]
Dietary iron (1 study)								
Dietary iron	Case-control, 2007-2014	113/226, mean age: 53.7, men: 0.0	106-item FFQ, amount	Upper vs. lower median (reference)	OR	1.00 (0.63, 1.57)	National Cancer Center	2016 [77]
Dietary calcium (1 study)								
Dietary calcium	Case-control, 2007-2014	113/226, mean age: 53.7, men: 0.0	106-item FFQ, amount	Upper vs. lower median (reference)	OR	0.55 (0.35, 0.89)	National Cancer Center	2016 [77]
Preservation and processing of foods								
Sodium (1 study)								
Sodium	Cohort, 2004-2008, median follow-up: 7.0	136/7,637, mean age: 48.4, men: 54.6	3-day DR, amount	≥4,000 vs. <4,000 mg/day (reference)	HR	1.11 (0.72, 1.69)	National Cancer Center	2014 [13]
Non-alcoholic drinks								
Coffee (1 study)								
Coffee	Cross-sectional, 2004-2016	1,410/160,810,mean age: 53.2, men: 34.3	106-item FFQ, frequency	>60 cups/mo vs. no drink (reference)	OR	0.71 (0.59, 0.85)	KoGES-HEXA	2021 [31]
Other dietary exposures								
Dietary pattern (1 study)								
Factor analysis: traditional balanced diet	Cross-sectional, 2004-2013	495/56,439, mean age: 53.6, men: 33.8	106-item FFQ, amount	≥70th vs. <70th percentile (reference)	OR	0.79 (0.60, 1.05)	KoGES-HEXA	2021 [79]
Prudent diet						1.45 (1.14, 1.83)		
Noodle/meat diet						0.67 (0.51, 0.89)		
Rice-based diet						0.84 (0.65, 1.08)		
Dietary retinol (1 study)								
Dietary retinol	Case-control, 2007-2014	113/226, mean age: 53.7, men: 0.0	106-item FFQ, amount	Upper vs. lower median (reference)	OR	0.95 (0.60, 1.52)	National Cancer Center	2016 [77]

OR, odds ratio; RR, relative risk; HR, hazard ratio; CI, confidence interval; Ref, reference number; FFQ, food frequency questionnaire; DR, dietary record; KoGES-HEXA, Korean Genome and Epidemiology Study-Health Examinee.

**Table 5.** Literature review on diet and cervical cancer in Korea

Dietary factors	Study design, enrollment year	Sample size (cases/controls, non-cases), age (yr)	Diet assessment, amount or frequency	Risk estimate			Sources	Year [Ref]
				Category	Type	Effect (95% CI)		
Whole grains, fruits, and vegetables								
Dietary fiber (1 study)								
Dietary fiber	Case-control, 2006-2010	229/729, mean age: 44.2	95-item FFQ, amount	Q5 vs. Q1 (reference)	OR	0.62 (0.37, 1.02)	6 university-affiliated hospitals in Korea (Korea, Yonsei, Chungnam, Gachon, Inha, and Ajou University)	2019 [80]
Carotenoid (3 studies)								
Dietary β-carotene	Case-control, 2006-2010	229/729, mean age: 44.2	95-item FFQ, amount	Q5 vs. Q1 (reference)	OR	0.66 (0.41, 1.06)	6 university-affiliated hospitals in Korea (Korea, Yonsei, Chungnam, Gachon, Inha, and Ajou University)	2019 [80]
Dietary vitamin C (2 studies)								
Dietary vitamin C	Case-control, 2006-2010	229/729, mean age: 44.2	95-item FFQ, amount	Q5 vs. Q1 (reference)	OR	0.57 (0.35, 0.92)	6 university-affiliated hospitals in Korea (Korea, Yonsei, Chungnam, Gachon, Inha, and Ajou University)	2019 [80]
Non-alcoholic drinks								
Coffee (1 study)								
Coffee	Cross-sectional, 2004-2016	689/105,921, mean age: 53.2	106-item FFQ, frequency	>60 cups/mo vs. no drink (reference)	OR	0.98 (0.75, 1.27)	KoGES-HEXA	2021 [31]
Tea (1 study)								
Tea	Case-control, 2006-2010	229/729, mean age: 44.2	95-item FFQ, amount	Q5 vs. Q1 (reference)	OR	1.33 (0.85, 2.06)	6 university-affiliated hospitals in Korea (Korea, Yonsei, Chungnam, Gachon, Inha, and Ajou University)	2019 [80]
Other dietary exposures								
Dietary pattern (1 study)								
Index-based: DII	Case-control, 2006-2010	229/729, mean age: 44.2	95-item FFQ, amount	Per 1 unit increase in DII	OR	1.12 (1.00, 1.24)	6 university-affiliated hospitals in Korea (Korea, Yonsei, Chungnam, Gachon, Inha, and Ajou University)	2019 [80]
Glycemic load (1 study)								
Glycemic index	Case-control, since 2006	221/670, mean age: 45.2	95-item FFQ, amount	Q5 vs. Q1 (reference)	OR	0.46 (0.17, 1.21)	8 university-affiliated hospitals in Korea (not specified)	2020 [81]
Glycemic load				Q5 vs. Q1 (reference)		0.50 (0.19, 1.30)		
Dietary retinol (2 studies)								
Dietary retinol	Case-control, 2006-2007	144/288, most frequent age range: 40-49	95-item FFQ, amount	Q4 vs. Q1 (reference)	OR	0.81 (0.45, 1.46)	6 university-affiliated hospitals in Korea (Korea, Yonsei, Chungnam, Gachon, Inha, and Ajou University)	2010 [82]

OR, odds ratio; RR, relative risk; HR, hazard ratio; CI, confidence interval; FFQ, food frequency questionnaire; DII, dietary inflammatory index; KoGES-HEXA, Korean Genome and Epidemiology Study-Health Examinee.

**Table 6.** Summary of the meta-analysis results on diet and cancer in Korea

Dietary exposure	Outcome	WCRF/AICR evidence level	No. of studies	Heterogeneity, I^2^ (%)	Model	Summary OR or RR (95% CI)	p for Egger’s test
Fruits and vegetables	Gastric cancer	-	7	82.2	Random-effects	0.59 (0.40, 0.86)	0.177
Fruits	Gastric cancer	Limited-suggestive, protective factor	5	54.7	Random-effects	0.72 (0.51, 1.01)	0.998
Vegetables	Gastric cancer	-	6	84.6	Random-effects	0.54 (0.32, 0.90)	0.227
Dietary vitamin C	Gastric cancer	-	4	0.0	Fixed-effect	0.74 (0.59, 0.92)	0.904
Pickled vegetables and kimchi	Gastric cancer	Probable, risk factor	7	91.6	Random-effects	1.31 (0.90, 1.90)	0.030
Salted seafood and fish	Gastric cancer	Probable, risk factor	4	59.2	Random-effects	0.96 (0.62, 1.51)	0.184
Fermented soy products	Gastric cancer	Probable, risk factor	4	56.3	Random-effects	1.56 (1.08, 2.27)	0.500
Meat	Gastric cancer	-	5	49.3	Fixed-effect	0.99 (0.92, 1.06)	0.599
Fruits and vegetables	Colorectal cancer	-	6	51.4	Random-effects	0.63 (0.49, 0.80)	0.665
Fruits	Colorectal cancer	Limited-suggestive, protective factor	4	23.2	Fixed-effect	0.69 (0.56, 0.86)	0.879
Vegetables	Colorectal cancer	Limited-suggestive, protective factor	5	62.4	Random-effects	0.58 (0.42, 0.80)	0.820
Meat	Colorectal cancer	-	8	77.8	Random-effects	1.35 (0.99, 1.85)	0.993
Red meat	Colorectal cancer	Probable, risk factor	5	84.8	Random-effects	1.39 (0.76, 2.57)	0.092
Fruits and vegetables	Breast cancer	-	5	77.0	Random-effects	0.72 (0.53, 0.98)	0.852
Fruits	Breast cancer	-	4	56.4	Random-effects	0.77 (0.55, 1.08)	0.988
Vegetables	Breast cancer	Limited-suggestive, protective factor	4	0.0	Fixed-effect	0.93 (0.78, 1.12)	0.599
Dietary carotenoids	Breast cancer	Limited-suggestive, protective factor	4	65.8	Random-effects	0.79 (0.55, 1.12)	0.264
Dietary vitamin C	Breast cancer	-	5	47.5	Fixed-effect	1.01 (0.98, 1.03)	0.331
Meat	Breast cancer	-	4	79.6	Random-effects	1.17 (0.83, 1.65)	0.583
Fish	Breast cancer	-	5	86.1	Random-effects	1.00 (0.66, 1.51)	0.116
Dairy products	Breast cancer	Limited-suggestive, protective factor	5	27.5	Fixed-effect	0.88 (0.76, 1.02)	0.567

WCRF/AICR, World Cancer Research Fund/American Institute for Cancer Research; OR, odds ratio; RR, relative risk; CI, confidence interval.

**Table 7.** Areas of focus for future epidemiological research on diet-cancer associations in Korea

Area of focus	Points
Study design	Longitudinal studies with sufficient statistical power are required to examine the temporal associations between diet and cancer risk
Cancer type	Further studies on anatomical sites with a substantial burden of disease that have been understudied in relation to dietary factors are suggested (e.g., lung, prostate, and liver) [2]
Confounder	Studies controlling for the major confounders with respect to specific cancer types should be considered
Attributable risk	To estimate the attributable risk of diet on cancer in Korean population, combining cohort studies that share dietary assessment methods and conducting pooled analyses are advised when examining diet-cancer associations to further estimate the cancer burden attributable to dietary factors
Life-course perspective	To consider the time-varying nature of nutrition, considering the role of diet during the early-life period, analyzing dietary pattern methods, and utilizing repeated measures of dietary assessment or recovery biomarkers of nutritional status are suggested [95]
Biological mechanism	To elucidate the biological mechanisms in diet-cancer research, further investigations of molecular subtypes of cancer and the interaction between diet and exposomes (e.g., environment, genomics, metabolomics, or gut microbiota profiles) are warranted [95]

## SUPPLEMENTARY MATERIALS

Supplement Material 1. Search terms used in each literature database

Supplement Material 2. Dietary exposures and cancer sites reviewed in this paper

Supplement Material 3. List of covariates for the research articles on diet and gastric cancer in Korea

Supplement Material 4. List of covariates for the research articles on diet and colorectal cancer in Korea

Supplement Material 5. List of covariates for the research articles on diet and breast cancer in Korea

Supplement Material 6. List of covariates for the research articles on diet and thyroid cancer in Korea

Supplement Material 7. List of covariates for the research articles on diet and cervical cancer in Korea

Supplement Material 8. Joanna Briggs Institute Critical Appraisal Checklist for Cohort Studies

Supplement Material 9. Quality assessment using the Joanna Briggs Institute (JBI) Critical Appraisal Checklist for Cohort Studies (n=10)

Supplement Material 10. Joanna Briggs Institute Critical Appraisal Tool for Case‒Control Studies

Supplement Material 11. Quality assessment using the Joanna Briggs Institute (JBI) Critical Appraisal Checklist for Case‒Control Studies (n=60)

Supplement Material 12. Joanna Briggs Institute Critical Appraisal Tool for Cross-Sectional Studies

Supplement Material 13. Quality assessment using the Joanna Briggs Institute (JBI) Critical Appraisal Checklist for Cross-Sectional Studies (n=2)

Supplement Material 14-1. Association between fruits and vegetables intake and the risk of gastric cancer in a random-effects model meta-analysis of observational studies (n=7)

Supplement Material 14-2. Begg’s funnel plot and Egger’s test for identifying publication bias in a meta-analysis of studies on the association between fruits and vegetables intake and the risk of gastric cancer (n=7)

Supplement Material 15-1. Association between fruits intake and the risk of gastric cancer in a random-effects model meta-analysis of observational studies (n=5)

Supplement Material 15-2. Begg’s funnel plot and Egger’s test for identifying publication bias in a meta-analysis of studies on the association between fruits intake and the risk of gastric cancer (n=5)

Supplement Material 16-1. Association between vegetables intake and the risk of gastric cancer in a random-effects model meta-analysis of observational studies (n=6)

Supplement Material 16-2. Begg’s funnel plot and Egger’s test for identifying publication bias in a meta-analysis of studies on the association between vegetables intake and the risk of gastric cancer (n=6)

Supplement Material 17-1. Association between dietary vitamin C intake and the risk of gastric cancer in a fixed-effect model meta-analysis of observational studies (n=4)

Supplement Material 17-2. Begg’s funnel plot and Egger’s test for identifying publication bias in a meta-analysis of studies on the association between dietary vitamin C intake and the risk of gastric cancer (n=4)

Supplement Material 18-1. Association between pickled vegetables and kimchi intake and the risk of gastric cancer in a random-effects model meta-analysis of observational studies (n=7)

Supplement Material 18-2. Begg’s funnel plot and Egger’s test for identifying publication bias in a meta-analysis of studies on the association between pickled vegetables and kimchi intake and the risk of gastric cancer (n=7)

Supplement Material 19-1. Association between salted seafood and fish intake and the risk of gastric cancer in a random-effects model meta-analysis of observational studies (n=4)

Supplement Material 19-2. Begg’s funnel plot and Egger’s test for identifying publication bias in a meta-analysis of studies on the association between salted seafood and fish intake and the risk of gastric cancer (n=4)

Supplement Material 20-1. Association between fermented soy products intake and the risk of gastric cancer in a random-effects model meta-analysis of observational studies (n=4)

Supplement Material 20-2. Begg’s funnel plot and Egger’s test for identifying publication bias in a meta-analysis of studies on the association between fermented soy products intake and the risk of gastric cancer (n=4)

Supplement Material 21-1. Association between meat intake and the risk of gastric cancer in a fixed-effect model meta-analysis of observational studies (n=5)

Supplement Material 21-2. Begg’s funnel plot and Egger’s test for identifying publication bias in a meta-analysis of studies on the association between meat intake and the risk of gastric cancer (n=5)

Supplement Material 22-1. Association between fruits and vegetables intake and the risk of colorectal cancer in a random-effects model meta-analysis of observational studies (n=6)

Supplement Material 22-2. Begg’s funnel plot and Egger’s test for identifying publication bias in a meta-analysis of studies on the association between fruits and vegetables intake and the risk of colorectal cancer (n=6)

Supplement Material 23-1. Association between fruits intake and the risk of colorectal cancer in a fixed-effect model meta-analysis of observational studies (n=4)

Supplement Material 23-2. Begg’s funnel plot and Egger’s test for identifying publication bias in a meta-analysis of studies on the association between fruits intake and the risk of colorectal cancer (n=4)

Supplement Material 24-1. Association between vegetables intake and the risk of colorectal cancer in a random-effects model meta-analysis of observational studies (n=5)

Supplement Material 24-2. Begg’s funnel plot and Egger’s test for identifying publication bias in a meta-analysis of studies on the association between vegetables intake and the risk of colorectal cancer (n=5)

Supplement Material 25-1. Association between meat intake and the risk of colorectal cancer in a random-effects model meta-analysis of observational studies (n=8)

Supplement Material 25-2. Begg’s funnel plot and Egger’s test for identifying publication bias in a meta-analysis of studies on the association between meat intake and the risk of colorectal cancer (n=8)

Supplement Material 26-1. Association between red meat intake and the risk of colorectal cancer in a random-effects model meta-analysis of observational studies (n=4)

Supplement Material 26-2. Begg’s funnel plot and Egger’s test for identifying publication bias in a meta-analysis of studies on the association between red meat intake and the risk of colorectal cancer (n=4)

Supplement Material 27-1. Association between fruits and vegetables intake and the risk of breast cancer in a random-effects model meta-analysis of observational studies (n=5)

Supplement Material 27-2. Begg’s funnel plot and Egger’s test for identifying publication bias in a meta-analysis of studies on the association between fruits and vegetables intake and the risk of breast cancer (n=5)

Supplement Material 28-1. Association between fruits intake and the risk of breast cancer in a random-effects model meta-analysis of observational studies (n=4)

Supplement Material 28-2. Begg’s funnel plot and Egger’s test for identifying publication bias in a meta-analysis of studies on the association between fruits intake and the risk of breast cancer (n=4)

Supplement Material 29-1. Association between vegetables intake and the risk of breast cancer in a fixed-effect model meta-analysis of observational studies (n=4)

Supplement Material 29-2. Begg’s funnel plot and Egger’s test for identifying publication bias in a meta-analysis of studies on the association between vegetables intake and the risk of breast cancer (n=4)

Supplement Material 30-1. Association between dietary carotenoids intake and the risk of breast cancer in a random-effects model meta-analysis of observational studies (n=4)

Supplement Material 30-2. Begg’s funnel plot and Egger’s test for identifying publication bias in a meta-analysis of studies on the association between dietary carotenoids intake and the risk of breast cancer (n=4)

Supplement Material 31-1. Association between dietary vitamin C intake and the risk of breast cancer in a fixed-effect model meta-analysis of observational studies (n=5)

Supplement Material 31-2. Begg’s funnel plot and Egger’s test for identifying publication bias in a meta-analysis of studies on the association between dietary vitamin C intake and the risk of breast cancer (n=5)

Supplement Material 32-1. Association between meat intake and the risk of breast cancer in a random-effects model meta-analysis of observational studies (n=4)

Supplement Material 32-2. Begg’s funnel plot and Egger’s test for identifying publication bias in a meta-analysis of studies on the association between meat intake and the risk of breast cancer (n=4)

Supplement Material 33-1. Association between fish intake and the risk of breast cancer in a random-effects model meta-analysis of observational studies (n=5)

Supplement Material 33-2. Begg’s funnel plot and Egger’s test for identifying publication bias in a meta-analysis of studies on the association between fish intake and the risk of breast cancer (n=5)

Supplement Material 34-1. Association between dairy products intake and the risk of breast cancer in a fixed-effect model meta-analysis of observational studies (n=5)

Supplement Material 34-2. Begg’s funnel plot and Egger’s test for identifying publication bias in a meta-analysis of studies on the association between dairy products intake and the risk of breast cancer (n=5)
