# Supplementary material for: Dietary intake and cancer incidence in Korean adults: a systematic review and meta-analysis of observational studies
Source: Epidemiol Health. 2023 Nov 30;45:e2023102. doi: 10.4178/epih.e2023102 (PMC10876448; doi:10.4178/epih.e2023102)
Supplement: Supplement Material 1. — Search terms used in each literature database [file epih-45-e2023102-Supplementary-1.docx]

**Supplementary Material 1.** Search terms used in each literature database

| **Database** | **Search term** | **Results** |
| --- | --- | --- |
| PubMed | (((diet[Title/Abstract]) or (food[Title/Abstract]) or (intake[Title/Abstract]) or (nutrition[Title/Abstract])) and ((korea) or (korean)) and (((cancer[Title/Abstract]) or (cancer[MeSH Terms])) and (risk[Title/Abstract]))) AND ("2000"[Date - Publication] : "2022"[Date - Publication]) and (humans[Filter]) and (English[Language]) | 646 |
| Embase | ((('diet'/exp OR 'diet':ab,ti) OR ('food'/exp OR 'food':ab,ti) OR ('intake'/exp OR 'intake':ab,ti) OR ('nutrition'/exp OR 'nutrition':ab,ti)) AND ('Korea' or 'Korean') AND (('cancer'/exp OR 'cancer':ab,ti) AND ('risk'/exp OR 'risk':ab,ti))) AND [2000-2022]/py AND [humans]/lim AND [english]/lim | 2308 |
| KoreaMed | ((("diet"[TIAB]) OR ("food"[TIAB]) OR ("intake"[TIAB]) OR ("nutrition"[TIAB])) AND ((("cancer"[TIAB]) OR (cancer[MH])) AND ("risk"[TIAB]))) AND (2000:2022[DPY]) | 316 |
