# Supplementary material for: Dietary intake and cancer incidence in Korean adults: a systematic review and meta-analysis of observational studies
Source: Epidemiol Health. 2023 Nov 30;45:e2023102. doi: 10.4178/epih.e2023102 (PMC10876448; doi:10.4178/epih.e2023102)

**Supplementary Material 2.** Dietary exposures and cancer sites reviewed in this paper. The contents are modified from the original summary of evidence table provided by the WCRF/AICR (Reference: World Cancer Research Fund International. Summary of evidence on diet, nutrition, physical activity, and the prevention of cancer [cited 2023 October 29]. Available from: https://www.wcrf.org/diet-activity-and-cancer/interactive-cancer-risk-matrix/). Numbers in the box indicate the counts of studies.


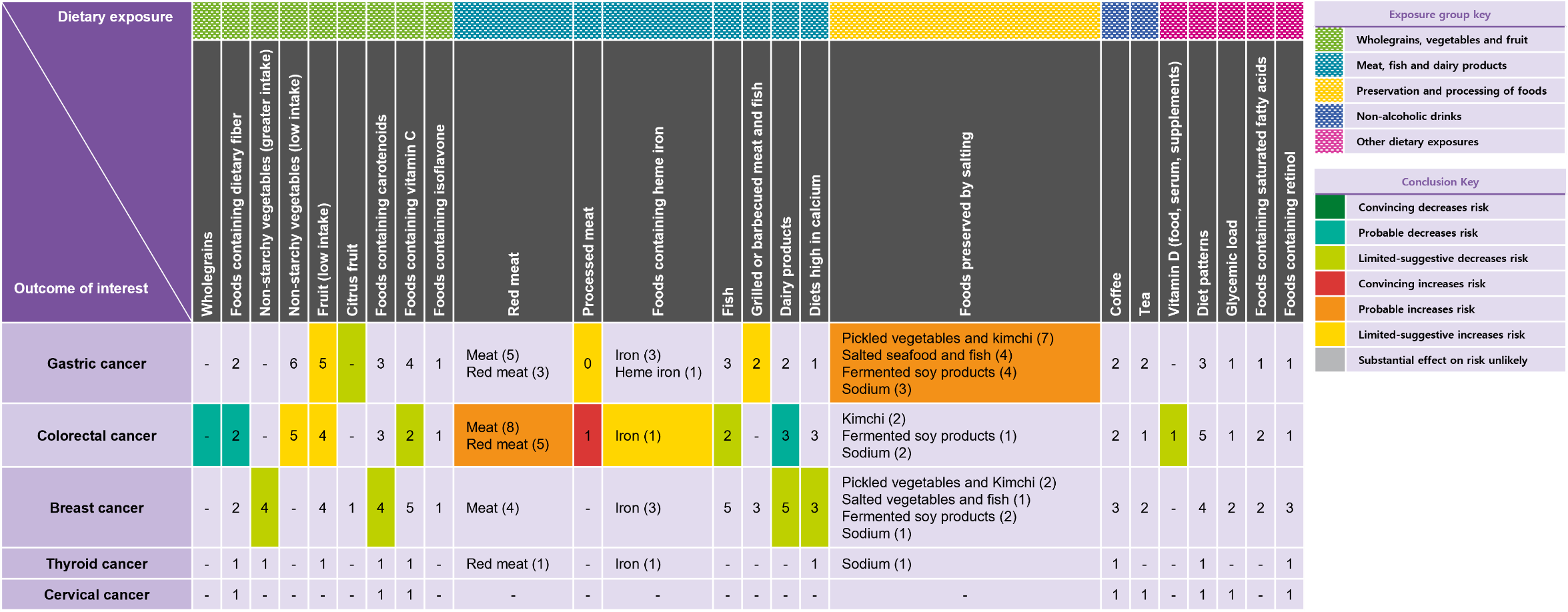

Supplement: Supplement Material 2. — Dietary exposures and cancer sites reviewed in this paper [file epih-45-e2023102-Supplementary-2.docx]
