# Supplementary material for: Dietary intake and cancer incidence in Korean adults: a systematic review and meta-analysis of observational studies
Source: Epidemiol Health. 2023 Nov 30;45:e2023102. doi: 10.4178/epih.e2023102 (PMC10876448; doi:10.4178/epih.e2023102)
Supplement: Supplement Material 3. — List of covariates for the research articles on diet and gastric cancer in Korea [file epih-45-e2023102-Supplementary-3.docx]

**Supplementary Material 3.** List of covariates for the research articles on diet and gastric cancer in Korea

| **Year, reference** | **Lists of covariates** | | | | | | |
| --- | --- | --- | --- | --- | --- | --- | --- |
|  | **Demographic characteristics** | **Socioeconomic status** | **Lifestyle factors** | **Anthropometry** | **Family history** | ***Helicobacter pylori* infection** | **Dietary factors and others** |
| 2022/ [11] | Age and gender | Education | Smoking, drinking, and physical activity | BMI | First-degree family history of GC | O | Sodium consumption |
| 2005/ [12] | Age and gender | A composite scale of education and income |  |  | First-degree family history of GC | O | Refrigerator use |
| 2014/ [13] | Age and gender | Education, income, and marital status | Smoking, drinking, and physical activity | BMI |  |  | Total energy intake |
| 2013/ [14] | Age, gender, and area of residence |  | Smoking, drinking | BMI |  |  |  |
| 2016/ [15] |  | Education, income, job | Smoking, physical activity |  | First-degree family history of GC | O |  |
| 2005/ [16] | Age and gender |  |  |  |  |  |  |
| 2003/ [17] | Age and gender | Education | Smoking, drinking |  | Family history of GC | O |  |
| 2002/ [18] | Age and gender | A composite scale of education and income |  |  | First-degree family history of GC |  | Refrigerator use |
| 2000/ [19] | - | - | - | - | - | - | - |
| 2022/ [20] | Age and gender | Education | Smoking ,drinking | BMI | Family history of GC |  | Total energy intake |
| 2018/ [21] | Age and gender | Education, income, job | Smoking, physical activity |  | First-degree family history of GC | O | Total energy intake |
| 2005/ [22] | Age and gender | Education |  |  |  |  | History of gastritis or gastric ulcer |
| 2017/ [23] |  | Education | Smoking ,drinking, and physical activity |  |  | O |  |
| 2010/ [24] | Age and gender |  | Smoking, drinking, and physical activity | BMI | Family history of cancer |  |  |
| 2002/ [25] | Age and gender |  |  |  |  | O |  |
| 2003/ [26] | - | - | - | - | - | - | - |
| 2021/ [27] | Age and gender | Education, income and job | Smoking and drinking | BMI | First-degree family history of GC | O |  |
| 2009/ [28] | Age and gender |  |  |  |  |  | Total energy intake |
| 2021/ [29] | Age and gender | Education | Smoking and drinking | BMI | First-degree family history of GC | O | Total energy intake |
| 2020/ [30] | Age, gender, and survey year |  | Smoking and drinking | BMI |  |  |  |
| 2021/ [31] | Age and gender | Income | Smoking, drinking, and physical activity | BMI |  |  | Histories of hypertension, diabetes, dyslipidemia, stroke, and ischemic heart diseases, and nutritional intake (total calories, protein, fat, and carbohydrate) |
| 2021/ [32] | Age and gender | Education, income, and job | Smoking and physical activity |  | First-degree family history of GC | O | Total energy intake and other dietary pattern |
| 2020/ [33] | Age | Education | Smoking and physical activity | BMI | First-degree family history of GC | O | Total energy intake |
| 2017/ [34] |  | Education | Smoking, drinking, and physical activity | BMI | First-degree family history of GC | O | Total energy intake |
| 2022/ [35] | Age, gender, and hospital | Education | Smoking and drinking | BMI | Family history of GC | O | Non-carbohydrate fruit, vegetable, and energy intake |

BMI: body mass index; GC: gastric cancer.
