# Supplementary material for: Dietary intake and cancer incidence in Korean adults: a systematic review and meta-analysis of observational studies
Source: Epidemiol Health. 2023 Nov 30;45:e2023102. doi: 10.4178/epih.e2023102 (PMC10876448; doi:10.4178/epih.e2023102)
Supplement: Supplement Material 4. — List of covariates for the research articles on diet and colorectal cancer in Korea [file epih-45-e2023102-Supplementary-4.docx]

**Supplementary Material 4.** List of covariates for the research articles on diet and colorectal cancer in Korea

| **Year, reference** | **Lists of covariates** | | | | | |
| --- | --- | --- | --- | --- | --- | --- |
|  | **Demographic characteristics** | **Socioeconomic status** | **Lifestyle factors** | **Anthropometry** | **Family history** | **Dietary factors and others** |
| 2015/ [36] | Age and gender | Education and income | Smoking, drinking, and physical activity | BMI |  | Total energy intake and dietary fiber intake |
| 2005/ [37] | Age and gender |  | Smoking and drinking |  |  | Total energy intake |
| 2017/ [38] | Age | Education | Drinking and physical activity | BMI |  | Total energy intake, red meat intake, and processed meat intake |
| 2014/ [13] | Age and gender | Education, income, and marital status | Smoking, drinking, and physical activity | BMI |  | Total energy intake |
| 2005/ [39] | (Stratified by gender)  Age | Education | Smoking and drinking |  |  | Total energy intake |
| 2003/ [40] | Age and gender |  | Smoking and drinking |  |  |  |
| 2019/ [41] | Age and gender | Education, income, and job | Drinking and physical activity | BMI | First-degree family history of CRC | Total energy intake |
| 2017/ [42] | Age and gender | Education and physical activity |  | BMI | First-degree family history of CRC | Total energy intake |
| 2011/ [43] | Age and gender |  | Smoking , drinking, and physical activity | BMI | Family history of cancer |  |
| 2006/ [44] | - | - | - | - | - | - |
| 2019/ [45] | Age and gender |  | Physical activity |  | Family history of CRC | History of diabetes and dairy intake |
| 2019/ [46] | Age and gender | Education | Smoking and drinking | BMI |  | Total energy intake and multivitamin supplement use |
| 2021/ [47] |  | Education | Smoking, drinking, and physical activity | BMI |  | Total energy intake, red meat intake, and processed meat intake |
| 2015/ [48] | (Stratified by gender)  Age | Education | Physical activity |  |  | Total energy intake, dietary fiber intake, and calcium supplement use |
| 2015/ [49] | (Stratified by gender)  Age | Education | Drinking and physical activity |  |  |  |
| 2021/ [50] | Age and gender | Education and job | Smoking, drinking, and physical activity | BMI | First-degree family history of CRC | Total energy intake and coffee additives |
| 2021/ [31] | Age and gender | Income | Smoking, drinking, and physical activity | BMI |  | Histories of hypertension, diabetes, hyperlipidemia, stroke, and ischemic heart disease, and nutritional intake (total calories, protein, fat, and carbohydrate) |
| 2019/ [51] |  | Education | Smoking, drinking, and physical activity | BMI | First-degree family history of CRC | Dietary inflammatory index |
| 2022/ [52] | Age and gender | Education | Smoking, drinking, and physical activity | BMI | First-degree family history of CRC | Total energy intake, comorbidity (any history of cancer, heart diseases, or diabetes), regular use of aspirin or other NSAIDs, and hormone replacement therapy (among females) |
| 2022/ [53] | Age and gender | Education, income, and job | Smoking, drinking, and physical activity | BMI | First-degree family history of CRC | Total energy intake and diabetes status |
| 2018/ [54] | Age and gender | Education | Physical activity |  | First-degree family history of CRC | Total energy intake |
| 2016/ [55] |  | Education, income, job, and marital status | Smoking, drinking, and physical activity | BMI | First-degree family history of CRC | Total energy intake |
| 2016/ [56] | Age and gender | Education | Physical activity | BMI | Family history of CRC | Total energy intake |
| 2022/ [57] | Age and gender | Education, income, and job | Smoking, drinking, and physical activity |  | First-degree family history of CRC | Total energy intake |

BMI: body mass index; CRC: colorectal cancer.
