# Supplementary material for: Dietary intake and cancer incidence in Korean adults: a systematic review and meta-analysis of observational studies
Source: Epidemiol Health. 2023 Nov 30;45:e2023102. doi: 10.4178/epih.e2023102 (PMC10876448; doi:10.4178/epih.e2023102)
Supplement: Supplement Material 5. — List of covariates for the research articles on diet and breast cancer in Korea [file epih-45-e2023102-Supplementary-5.docx]

**Supplementary Material 5.** List of covariates for the research articles on diet and breast cancer in Korea

| **Year, reference** | **Lists of covariates** | | | | | | |
| --- | --- | --- | --- | --- | --- | --- | --- |
|  | **Demographic characteristics** | **Socioeconomic status** | **Lifestyle factors** | **Anthropometry** | **Family history** | **Reproductive or hormone related factors** | **Dietary factors and others** |
| 2008/ [58] | Residence | Occupation |  | BMI | Family history of BC | Delivery, miscarriage, breast feeding ,periods of breastfeeding, hormone replacement therapy | Total energy intake |
| 2000/ [59] | Age | Income |  |  | Family history of BC | Age at menarche and pregnancy |  |
| 2017/ [60] | Age |  | Smoking, drinking, and physical activity | BMI | Family history of BC | Age at menarche, parity, oral contraceptive use, benign breast tumor history, hormone use and menopausal status, and age at menopause |  |
| 2010/ [61] | Age | Education and job | Smoking, drinking, and physical activity | BMI | Family history of BC | Menopausal status, age at menarche, and parity | Total energy intake and current use of dietary supplements |
| 2007/ [62] | Age | Education and income | Smoking, drinking, and physical activity |  | First-degree family history of BC | Age at menarche, parity, age at first live birth, history of breastfeeding, and use of hormones (oral contraceptives and hormone replacement) |  |
| 2007/ [63] | Residence | Job |  | BMI | Family history of BC | Delivery, miscarriage, breast feeding, periods of breast feeding, and hormone replacement therapy |  |
| 2003/ [64] | Age |  |  | BMI | Family history of BC |  |  |
| 2012/ [65] | Age | Education |  | BMI | First-and second-degree family history of BC | Age of first full-term pregnancy | Total energy intake |
| 2010/ [66] |  |  |  |  | Family history of BC. | Number of children, breastfeeding, and year of first childbirth | Multivitamin supplement use and intake of soy protein, mushroom, and dietary fat |
| 2003/ [67] |  |  |  | BMI | First-degree family history of BC | Age at menarche, total menstrual periods, pregnancy, total number of full-term deliveries, and total periods of breastfeeding |  |
| 2022/ [68] | Age |  | Smoking, drinking, and physical activity | BMI | Family history of BC | Diagnosis of benign BC, age at menarche, menopausal status, menopausal age, and breastfeeding | Total energy intake |
| 2010/ [69] | Age | Education and job | Smoking, drinking, and physical activity | BMI | Family history of BC | Menopausal status, age at menarche, parity, and postmenopausal hormone use for postmenopausal women | Total energy intake and current use of dietary supplements |
| 2009/ [70] | Age | Education and job | Smoking, drinking, and physical activity | BMI | Family history of BC | Parity, menopausal status, and age at menarche | Total energy intake and supplement use |
| 2020/ [71] |  | Education | Drinking and physical activity | BMI | Family history of BC | Parity, age at first birth, age at menarche, and oral contraceptive use | Total energy intake |
| 2021/ [31] | Age | Income | Drinking and physical activity | BMI |  |  | Histories of hypertension, diabetes, hyperlipidemia, stoke, or ischemic heart disease, and nutritional intake (total calories, protein, fat, and carbohydrate) |
| 2020/ [72] |  | Education | Drinking and physical activity | BMI | Family history of BC | Parity, age at first birth, age at menarche, and oral contraceptive use | Total energy intake |
| 2019/ [73] |  | Education and job |  | BMI and Height |  | Age at menarche and parity, | Total energy intake |
| 2013/ [74] | Age | Education | Smoking, drinking, and physical activity | BMI | Family history of BC | Age at menarche, parity, menopausal status, and postmenopausal hormone use for postmenopausal women | Total energy intake and dietary fiber intake |
| 2010/ [75] | Age. | Education and job | Smoking, drinking, and physical activity | BMI | Family history of BC | Menopausal status, age at menarche, parity, and postmenopausal hormone use for postmenopausal women | Total energy intake and current use of dietary supplements |
| 2010/ [76] |  | Drinking |  | BMI |  | Number of children and breastfeeding | Multivitamin supplement use and dietary factors including soy protein, folate, vitamin E, and fiber |

BMI: body mass index; BC: breast cancer.
