# Supplementary material for: Dietary intake and cancer incidence in Korean adults: a systematic review and meta-analysis of observational studies
Source: Epidemiol Health. 2023 Nov 30;45:e2023102. doi: 10.4178/epih.e2023102 (PMC10876448; doi:10.4178/epih.e2023102)
Supplement: Supplement Material 6. — List of covariates for the research articles on diet and thyroid cancer in Korea [file epih-45-e2023102-Supplementary-6.docx]

**Supplementary Material 6.** List of covariates for the research articles on diet and thyroid cancer in Korea

| **Year, reference** | **Lists of covariates** | | | | | |
| --- | --- | --- | --- | --- | --- | --- |
|  | **Demographic characteristics** | **Socioeconomic status** | **Lifestyle factors** | **Anthropometry** | **Reproductive factors (women)** | **Dietary factors and others** |
| 2016/ [77] |  |  | Smoking | BMI |  |  |
| 2014/ [13] |  | Education, income, and marital status | Smoking, drinking, and physical activity | BMI |  | Total energy intake |
| 2013/ [78] |  |  |  |  | Parity | Total energy intake and dietary sodium and vitamin E intake |
| 2021/ [31] | Age and gender | Income | Smoking, drinking, and physical activity | BMI |  | Histories of hypertension, diabetes mellitus, hyperlipidemia, stoke, or ischemic heart disease, and nutritional intake (total calories, protein, fat, and carbohydrate) |
| 2021/ [79] | Age, gender, residence area, and survey year | Education and income | Smoking, drinking, and physical activity | BMI | Menopause and initial menstruation | Total energy intake, fat intake (%), and carbohydrate intake (%) |

BMI: body mass index.
