# Supplementary material for: Dietary intake and cancer incidence in Korean adults: a systematic review and meta-analysis of observational studies
Source: Epidemiol Health. 2023 Nov 30;45:e2023102. doi: 10.4178/epih.e2023102 (PMC10876448; doi:10.4178/epih.e2023102)
Supplement: Supplement Material 7. — List of covariates for the research articles on diet and cervical cancer in Korea [file epih-45-e2023102-Supplementary-7.docx]

**Supplementary Material 7.** List of covariates for the research articles on diet and cervical cancer in Korea

| **Year, reference** | **Lists of covariates** | | | | | | |
| --- | --- | --- | --- | --- | --- | --- | --- |
|  | **Demographic characteristics** | **Socioeconomic status** | **Lifestyle factors** | **Anthropometry** | **Family history** | **Reproductive or hormone related factors** | **Dietary factors and others** |
| 2019/ [80] | Age | Education and marital status | Drinking | BMI |  | Pregnancy, oral contraceptive use, and menopausal status |  |
| 2021/ [31] | Age | Income | Smoking, drinking, and physical activity | BMI |  |  | Histories of hypertension, diabetes, hyperlipidemia, stroke, or ischemic heart disease, and nutritional intake (total calories, protein, fat, and carbohydrate) |
| 2020/ [81] | Age and hospitals | Education and marital status | Smoking and physical activity |  | Family history of CC | History of pregnancy and oral contraceptive use | Total energy intake |
| 2010/ [82] | Age | Smoking, drinking, and physical activity |  | BMI | Family history of CC | Human papillomavirus infection status |  |

BMI: body mass index; CC: cervical cancer.
