# Supplementary material for: Dietary intake and cancer incidence in Korean adults: a systematic review and meta-analysis of observational studies
Source: Epidemiol Health. 2023 Nov 30;45:e2023102. doi: 10.4178/epih.e2023102 (PMC10876448; doi:10.4178/epih.e2023102)
Supplement: Supplement Material 8. — Joanna Briggs Institute Critical Appraisal Checklist for Cohort Studies [file epih-45-e2023102-Supplementary-8.docx]

**Supplementary Material 8.** Joanna Briggs Institute Critical Appraisal Checklist for Cohort Studies [10]

| **JBI Checklist no.** |  |
| --- | --- |
| Q1 | Were the two groups similar and recruited from the same population? |
| Q2 | Were the exposures measured similarly to assign people to both exposed and unexposed groups? |
| Q3 | Was the exposure measured in a valid and reliable way? |
| Q4 | Were confounding factors identified? |
| Q5 | Were strategies to deal with confounding factors stated? |
| Q6 | Were the groups/participants free of the outcome at the start of the study (or at the moment of exposure)? |
| Q7 | Were the outcomes measured in a valid and reliable way? |
| Q8 | Was the follow up time reported and sufficient to be long enough for outcomes to occur? |
| Q9 | Was follow up complete, and if not, were the reasons to loss to follow up described and explored? |
| Q10 | Were strategies to address incomplete follow up utilized? |
| Q11 | Was appropriate statistical analysis used? |

Answers: yes, no, unclear or not applicable (N/A).
