# Supplementary material for: Dietary intake and cancer incidence in Korean adults: a systematic review and meta-analysis of observational studies
Source: Epidemiol Health. 2023 Nov 30;45:e2023102. doi: 10.4178/epih.e2023102 (PMC10876448; doi:10.4178/epih.e2023102)
Supplement: Supplement Material 9. — Quality assessment using the Joanna Briggs Institute (JBI) Critical Appraisal Checklist for Cohort Studies (n=10) [file epih-45-e2023102-Supplementary-9.docx]

**Supplementary Material 9.** Quality assessment using the Joanna Briggs Institute (JBI) Critical Appraisal Checklist for Cohort Studies (n=10)

| **JBI Checklist no.** | **Joanna Briggs Institute Critical Appraisal Checklist for Cohort Studies** | | | | | | | | | | | |
| --- | --- | --- | --- | --- | --- | --- | --- | --- | --- | --- | --- | --- |
| **Author, year** | **Q1** | **Q2** | **Q3** | **Q4** | **Q5** | **Q6** | **Q7** | **Q8** | **Q9** | **Q10** | **Q11** | **Quality category** |
| Wie, 2014 [13] | Yes | Yes | Yes | Yes | Yes | Yes | Yes | Yes | Yes | N/A | Yes | High (90.9%) |
| Ko, 2013 [14] | Yes | Yes | N/A | Yes | Yes | Yes | Yes | Yes | Yes | N/A | Yes | High (81.8%) |
| Kim, 2010 [24] | Yes | Yes | N/A | Yes | Yes | Yes | Yes | Yes | Yes | N/A | Yes | High (81.8%) |
| Yoo, 2020 [30] | Yes | Yes | N/A | Yes | Yes | Yes | Yes | Yes | Yes | N/A | Yes | High (81.8%) |
| Kim, 2011 [43] | Yes | Yes | N/A | Yes | Yes | Yes | Yes | Yes | Yes | N/A | Yes | High (81.8%) |
| Lee, 2021 [47] | Yes | Yes | Yes | Yes | Yes | Yes | Yes | Yes | Yes | N/A | Yes | High (90.9%) |
| Song, 2022 [68] | Yes | Yes | Yes | Yes | Yes | Yes | Unclear | Yes | Yes | N/A | Yes | High (81.8%) |
| Kim, 2017 [60] | Yes | Yes | N/A | Yes | Yes | Yes | Yes | Yes | Yes | N/A | Yes | High (81.8%) |
| Shin, 2020 [71] | Yes | Yes | Yes | Yes | Yes | Yes | Yes | Yes | Yes | N/A | Yes | High (90.9%) |
| Shin, 2020 [72] | Yes | Yes | Yes | Yes | Yes | Yes | Yes | Yes | Yes | N/A | Yes | High (90.9%) |
| Criterion score (%) | 100 | 100 | 50 | 100 | 100 | 100 | 90 | 100 | 100 | N/A | 100 | - |

The criterion score is calculated by dividing the number of studies meeting one criterion by the total number of studies; Yes: meet the methodological quality criterion; No: not meet the methodological quality criterion; unclear: unclear about the methodological quality criterion; N/A: not applicable.
