# Supplementary material for: Dietary intake and cancer incidence in Korean adults: a systematic review and meta-analysis of observational studies
Source: Epidemiol Health. 2023 Nov 30;45:e2023102. doi: 10.4178/epih.e2023102 (PMC10876448; doi:10.4178/epih.e2023102)
Supplement: Supplement Material 10. — Joanna Briggs Institute Critical Appraisal Tool for Case‒Control Studies [file epih-45-e2023102-Supplementary-10.docx]

**Supplementary Material 10.** Joanna Briggs Institute Critical Appraisal Tool for Case‒Control Studies [10]

| **JBI Checklist no.** |  |
| --- | --- |
| Q1 | Were the groups comparable other than the presence of disease in cases or the absence of disease in controls? |
| Q2 | Were cases and controls matched appropriately? |
| Q3 | Were the same criteria used for identification of cases and controls? |
| Q4 | Was exposure measured in a standard, valid and reliable way? |
| Q5 | Was exposure measured in the same way for cases and controls? |
| Q6 | Were confounding factors identified? |
| Q7 | Were strategies to deal with confounding factors stated? |
| Q8 | Were outcomes assessed in a standard, valid and reliable way for cases and controls? |
| Q9 | Was the exposure period of interest long enough to be meaningful? |
| Q10 | Was appropriate statistical analysis used? |

Answers: yes, no, unclear or not applicable (N/A).
