# Supplementary material for: Dietary intake and cancer incidence in Korean adults: a systematic review and meta-analysis of observational studies
Source: Epidemiol Health. 2023 Nov 30;45:e2023102. doi: 10.4178/epih.e2023102 (PMC10876448; doi:10.4178/epih.e2023102)
Supplement: Supplement Material 11. — Quality assessment using the Joanna Briggs Institute (JBI) Critical Appraisal Checklist for Case‒Control Studies (n=60) [file epih-45-e2023102-Supplementary-11.docx]

**Supplementary Material 11.** Quality assessment using the Joanna Briggs Institute (JBI) Critical Appraisal Checklist for Case‒Control Studies (n=60)

| **JBI Checklist no.** | **Joanna Briggs Institute Critical Appraisal Checklist for Case-Control Studies** | | | | | | | | | | |
| --- | --- | --- | --- | --- | --- | --- | --- | --- | --- | --- | --- |
| **Author, year** | **Q1** | **Q2** | **Q3** | **Q4** | **Q5** | **Q6** | **Q7** | **Q8** | **Q9** | **Q10** | **Quality category** |
| Tran, 2022 [11] | Yes | Yes | Yes | Yes | Yes | Yes | Yes | Yes | Yes | Yes | High (100%) |
| Kim, 2005 [12] | Yes | Yes | Yes | Yes | Yes | Yes | Yes | Yes | Yes | Yes | High (100%) |
| Hoang, 2016 [15] | Yes | Yes | Yes | Yes | Yes | Yes | Yes | Yes | Yes | Yes | High (100%) |
| Nan, 2005 [16] | Yes | Yes | Yes | Yes | Yes | Unclear | Unclear | Yes | Yes | Unclear | Low (70%) |
| Lee, 2003 [17] | Yes | No | Yes | Yes | Yes | Yes | Yes | Yes | Yes | Yes | High (90%) |
| Kim, 2002 [18] | Yes | Yes | Yes | Yes | Yes | Yes | Yes | Yes | Yes | Yes | High (100%) |
| Park, 2000 [19] | Yes | Yes | Yes | Yes | Yes | No | No | Yes | Yes | Unclear | Low (70%) |
| Kim, 2022 [20] | Yes | Yes | Yes | Yes | Yes | Yes | Yes | Yes | Yes | Yes | High (100%) |
| Kim, 2018 [21] | Yes | Yes | Yes | Yes | Yes | Yes | Yes | Yes | Yes | Yes | High (100%) |
| Kim, 2005 [22] | Yes | Yes | Yes | N/A | Yes | Yes | Yes | Yes | Yes | Yes | High (90%) |
| Yang, 2017 [23] | Yes | Yes | Yes | Yes | Yes | Yes | Yes | Yes | Yes | Yes | High (100%) |
| Lee, 2002 [25] | Yes | No | Yes | Yes | Yes | Unclear | Unclear | Yes | Yes | Unclear | Low (60%) |
| Yun, 2003 [26] | Yes | Yes | Yes | Yes | Yes | No | No | Yes | Yes | Unclear | Low (70%) |
| Tran, 2021 [27] | Yes | Yes | Yes | Yes | Yes | Yes | Yes | Yes | Yes | Yes | High (100%) |
| Zhang, 2009 [28] | Yes | Yes | Yes | Yes | Yes | Unclear | Unclear | Yes | Yes | Unclear | Low (70%) |
| Kwak, 2021 [29] | Yes | Yes | Yes | Yes | Yes | Yes | Yes | Yes | Yes | Yes | High (100%) |
| Kim, 2021 [32] | Yes | Yes | Yes | Yes | Yes | Yes | Yes | Yes | Yes | Yes | High (100%) |
| Kim, 2020 [33] | Yes | Yes | Yes | Yes | Yes | Yes | Yes | Yes | Yes | Yes | High (100%) |
| Lee, 2017 [34] | Yes | Yes | Yes | Yes | Yes | Yes | Yes | Yes | Yes | Yes | High (100%) |
| Kim, 2022 [35] | Yes | Yes | Yes | Yes | Yes | Yes | Yes | Yes | Yes | Yes | High (100%) |
| Chun, 2015 [36] | Yes | No | Yes | Yes | Yes | Yes | Yes | Yes | Yes | Yes | High (90%) |
| Oh, 2015 [37] | Yes | No | Yes | Yes | Yes | Yes | Yes | Yes | Yes | Yes | High (90%) |
| Lee, 2017 [38] | Yes | Yes | Yes | Yes | Yes | Yes | Yes | Yes | Yes | Yes | High (100%) |
| Lee, 2005 [39] | Yes | No | Yes | Yes | Yes | Yes | Yes | Yes | Yes | Yes | High (90%) |
| Kim, 2003 [40] | Yes | Yes | Yes | N/A | Yes | No | No | Yes | N/A | Yes | Low (60%) |
| Kim, 2019 [41] | Yes | Yes | Yes | Yes | Yes | Yes | Yes | Yes | Yes | Yes | High (100%) |
| Cho, 2017 [42] | Yes | Yes | Yes | Yes | Yes | Yes | Yes | Yes | Yes | Yes | High (100%) |
| Ahn, 2006 [44] | Yes | No | Yes | N/A | Yes | No | No | Yes | Yes | Unclear | Low (50%) |
| Song, 2019 [45] | Yes | Yes | Yes | Yes | Yes | Yes | Yes | Yes | Yes | Yes | High (100%) |
| Kim, 2019 [46] | Yes | No | Yes | Yes | Yes | Yes | Yes | Yes | Yes | Yes | High (90%) |
| Han, 2015 [48] | Yes | Yes | Yes | Yes | Yes | Yes | Yes | Yes | Yes | Yes | High (100%) |
| Shin, 2015 [49] | Yes | Yes | Yes | Yes | Yes | Yes | Yes | Yes | Yes | Yes | High (100%) |
| Kim, 2021 [50] | Yes | Yes | Yes | Yes | Yes | Yes | Yes | Yes | Yes | Yes | High (100%) |
| Kim, 2019 [51] | Yes | Yes | Yes | Yes | Yes | Yes | Yes | Yes | Yes | Yes | High (100%) |
| Jun, 2022 [52] | Yes | Yes | Yes | Yes | Yes | Yes | Yes | Yes | Yes | Yes | High (100%) |

**Supplementary Material 11.** Cont’d.

| **JBI Checklist no.** | **Joanna Briggs Institute Critical Appraisal Checklist for Case-Control Studies** | | | | | | | | | | |
| --- | --- | --- | --- | --- | --- | --- | --- | --- | --- | --- | --- |
| **Author, year** | **Q1** | **Q2** | **Q3** | **Q4** | **Q5** | **Q6** | **Q7** | **Q8** | **Q9** | **Q10** | **Quality category** |
| Kim, 2022 [53] | Yes | Yes | Yes | Yes | Yes | Yes | Yes | Yes | Yes | Yes | High (100%) |
| Cho, 2018 [54] | Yes | Yes | Yes | Yes | Yes | Yes | Yes | Yes | Yes | Yes | High (100%) |
| Park, 2016 [55] | Yes | Yes | Yes | Yes | Yes | Yes | Yes | Yes | Yes | Yes | High (100%) |
| Cho, 2016 [56] | Yes | Yes | Yes | Yes | Yes | Yes | Yes | Yes | Yes | Yes | High (100%) |
| Lu, 2022 [57] | Yes | Yes | Yes | Yes | Yes | Yes | Yes | Yes | Yes | Yes | High (100%) |
| Lee, 2008 [58] | Yes | Yes | Unclear | Yes | Yes | Yes | Yes | Yes | Yes | Yes | High (90%) |
| Do, 2000 [59] | Yes | Yes | Yes | No | Yes | Yes | Yes | Yes | Yes | Yes | High (90%) |
| Yu, 2010 [61] | Yes | Yes | Yes | Yes | Yes | Yes | Yes | Yes | Yes | Yes | High (100%) |
| Do, 2007 [62] | Yes | Yes | Yes | Yes | Yes | Yes | Yes | Yes | Yes | Yes | High (100%) |
| Lee, 2007 [63] | Yes | Yes | Unclear | Yes | Yes | Yes | Yes | Yes | Yes | Yes | High (90%) |
| Lee, 2003 [64] | Yes | Unclear | Yes | Yes | Yes | Yes | Yes | Yes | Yes | Yes | High (90%) |
| Lee, 2012 [65] | Yes | Yes | Yes | Yes | Yes | Yes | Yes | Yes | Yes | Yes | High (100%) |
| Yang, 2010 [66] | Yes | Yes | Yes | Yes | Yes | Yes | Yes | Yes | Yes | Yes | High (100%) |
| Do, 2003 [67] | Yes | Yes | Yes | Yes | Yes | Yes | Yes | Yes | Yes | Yes | High (100%) |
| Kim, 2009 [70] | Yes | Yes | Yes | Yes | Yes | Yes | Yes | Yes | Yes | Yes | High (100%) |
| Cho, 2010 [69] | Yes | Yes | Yes | Yes | Yes | Yes | Yes | Yes | Yes | Yes | High (100%) |
| Lee, 2019 [73] | Yes | Yes | Yes | Yes | Yes | Yes | Yes | Yes | Yes | Yes | High (100%) |
| Woo, 2013 [74] | Yes | Yes | Yes | Yes | Yes | Yes | Yes | Yes | Yes | Yes | High (100%) |
| Cho, 2010 [75] | Yes | Yes | Yes | Yes | Yes | Yes | Yes | Yes | Yes | Yes | High (100%) |
| Yun, 2010 [76] | Yes | Yes | Yes | Yes | Yes | Yes | Yes | Yes | Yes | Yes | High (100%) |
| Cho, 2016 [77] | Yes | Yes | Yes | Yes | Yes | Yes | Yes | Yes | Yes | Yes | High (100%) |
| Jung, 2013 [78] | Yes | Yes | Yes | Yes | Yes | Yes | Yes | Yes | Yes | Yes | High (100%) |
| Sreeja, 2019 [80] | Yes | No | Yes | Yes | Yes | Yes | Yes | Yes | Yes | Yes | High (90%) |
| Sreeja, 2020 [81] | Yes | No | Yes | Yes | Yes | Yes | Yes | Yes | Yes | Yes | High (90%) |
| Kim, 2010 [82] | Yes | Yes | Yes | Yes | Yes | Yes | Yes | Yes | Yes | Yes | High (100%) |
| Criterion Score % | 100 | 83.3 | 96.7 | 93.3 | 100 | 88.3 | 88.3 | 100 | 98.3 | 90.0 | - |

The criterion score is calculated by dividing the number of studies meeting one criterion by the total number of studies; Yes: meet the methodological quality criterion; No: not meet the methodological quality criterion; unclear: unclear about the methodological quality criterion; N/A: not applicable.
