# Supplementary material for: Dietary intake and cancer incidence in Korean adults: a systematic review and meta-analysis of observational studies
Source: Epidemiol Health. 2023 Nov 30;45:e2023102. doi: 10.4178/epih.e2023102 (PMC10876448; doi:10.4178/epih.e2023102)
Supplement: Supplement Material 12. — Joanna Briggs Institute Critical Appraisal Tool for Cross-Sectional Studies [file epih-45-e2023102-Supplementary-12.docx]

**Supplementary Material 12.** Joanna Briggs Institute Critical Appraisal Tool for Cross-Sectional Studies [10]

| **JBI Checklist no.** |  |
| --- | --- |
| Q1 | Were the criteria for inclusion in the sample clearly defined? |
| Q2 | Were the study subjects and the setting described in detail? |
| Q3 | Was the exposure measured in a valid and reliable way? |
| Q4 | Were objective, standard criteria used for measurement of the condition? |
| Q5 | Were confounding factors identified? |
| Q6 | Were strategies to deal with confounding factors stated? |
| Q7 | Were the outcomes measured in a valid and reliable way? |
| Q8 | Was appropriate statistical analysis used? |

Answers: yes, no, unclear or not applicable (N/A).
