# Supplementary material for: Dietary intake and cancer incidence in Korean adults: a systematic review and meta-analysis of observational studies
Source: Epidemiol Health. 2023 Nov 30;45:e2023102. doi: 10.4178/epih.e2023102 (PMC10876448; doi:10.4178/epih.e2023102)
Supplement: Supplement Material 13. — Quality assessment using the Joanna Briggs Institute (JBI) Critical Appraisal Checklist for Cross-Sectional Studies (n=2) [file epih-45-e2023102-Supplementary-13.docx]

**Supplementary Material 13.** Quality assessment using the Joanna Briggs Institute (JBI) Critical Appraisal Checklist for Cross-Sectional Studies (n=2)

| **JBI Checklist no.** | **Joanna Briggs Institute Critical Appraisal Checklist for Cross-Sectional Studies** | | | | | | | | |
| --- | --- | --- | --- | --- | --- | --- | --- | --- | --- |
| **Author, year** | **Q1** | **Q2** | **Q3** | **Q4** | **Q5** | **Q6** | **Q7** | **Q8** | **Quality category** |
| Kim, 2021 [31] | Yes | Yes | Yes | Yes | Yes | Yes | Unclear | Yes | High (87.5%) |
| Song, 2021 [79] | Yes | Yes | Yes | Yes | Yes | Yes | Unclear | Yes | High (87.5%) |
| Criterion Score % | 100 | 100 | 100 | 100 | 100 | 100 | 0 | 100 | - |

The criterion score is calculated by dividing the number of studies meeting one criterion by the total number of studies; Yes: meet the methodological quality criterion; No: not meet the methodological quality criterion; unclear: unclear about the methodological quality criterion; N/A: not applicable.
