# Supplementary material for: Dietary intake and cancer incidence in Korean adults: a systematic review and meta-analysis of observational studies
Source: Epidemiol Health. 2023 Nov 30;45:e2023102. doi: 10.4178/epih.e2023102 (PMC10876448; doi:10.4178/epih.e2023102)

**Supplementary Material 14-1.** Association between fruits and vegetables intake and the risk of gastric cancer in a random-effects model meta-analysis of observational studies (n=7). ^a^ OR, odds ratio; RR, relative risk; HR, hazard ratio; CI, confidence interval.


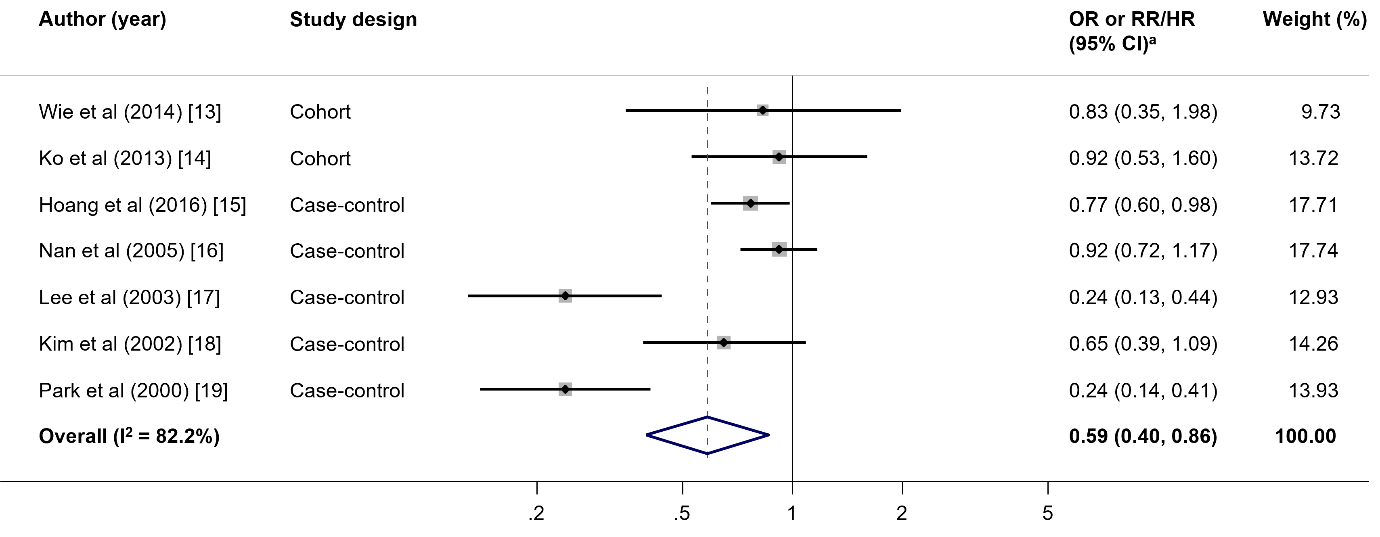

Supplement: Supplement Material 14-1. — Association between fruits and vegetables intake and the risk of gastric cancer in a random-effects model meta-analysis of observational studies (n=7) [file epih-45-e2023102-Supplementary-14-1.docx]
