# Supplementary material for: Dietary intake and cancer incidence in Korean adults: a systematic review and meta-analysis of observational studies
Source: Epidemiol Health. 2023 Nov 30;45:e2023102. doi: 10.4178/epih.e2023102 (PMC10876448; doi:10.4178/epih.e2023102)
Supplement: Supplement Material 24-1. — Association between vegetables intake and the risk of colorectal cancer in a random-effects model meta-analysis of observational studies (n=5) [file epih-45-e2023102-Supplementary-24-1.docx]

**Supplementary Material 24-1.** Association between vegetables intake and the risk of colorectal cancer in a random-effects model meta-analysis of observational studies (n=5). ^a^ OR, odds ratio; CI, confidence interval.

**
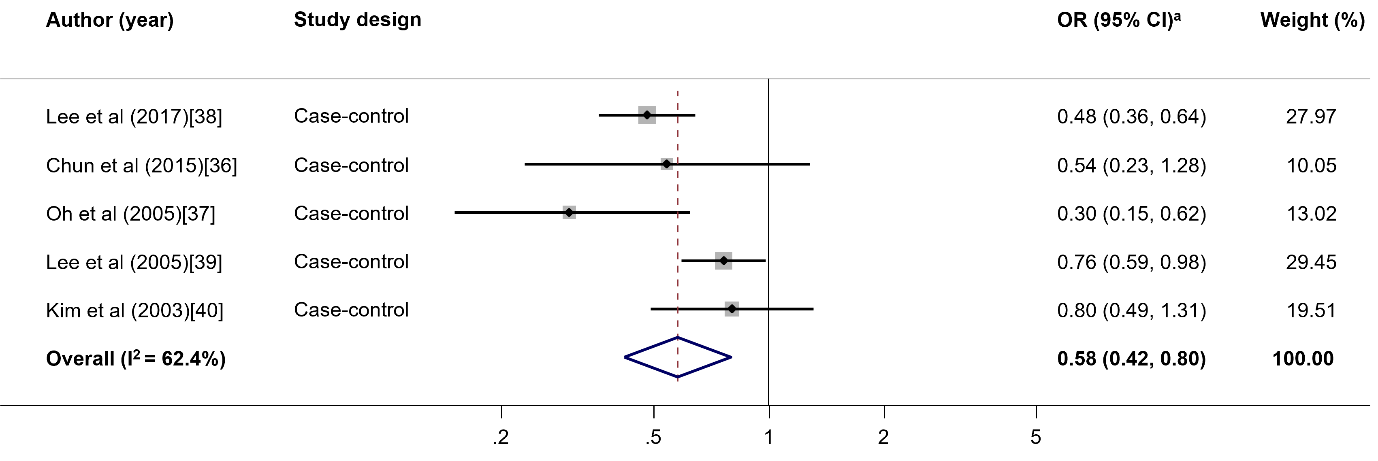
**
