# Supplementary material for: Dietary intake and cancer incidence in Korean adults: a systematic review and meta-analysis of observational studies
Source: Epidemiol Health. 2023 Nov 30;45:e2023102. doi: 10.4178/epih.e2023102 (PMC10876448; doi:10.4178/epih.e2023102)

**Supplementary Material 25-1.** Association between meat intake and the risk of colorectal cancer in a random-effects model meta-analysis of observational studies (n=8). ^a^ OR, odds ratio; RR, relative risk; HR, hazard ratio; CI, confidence interval.


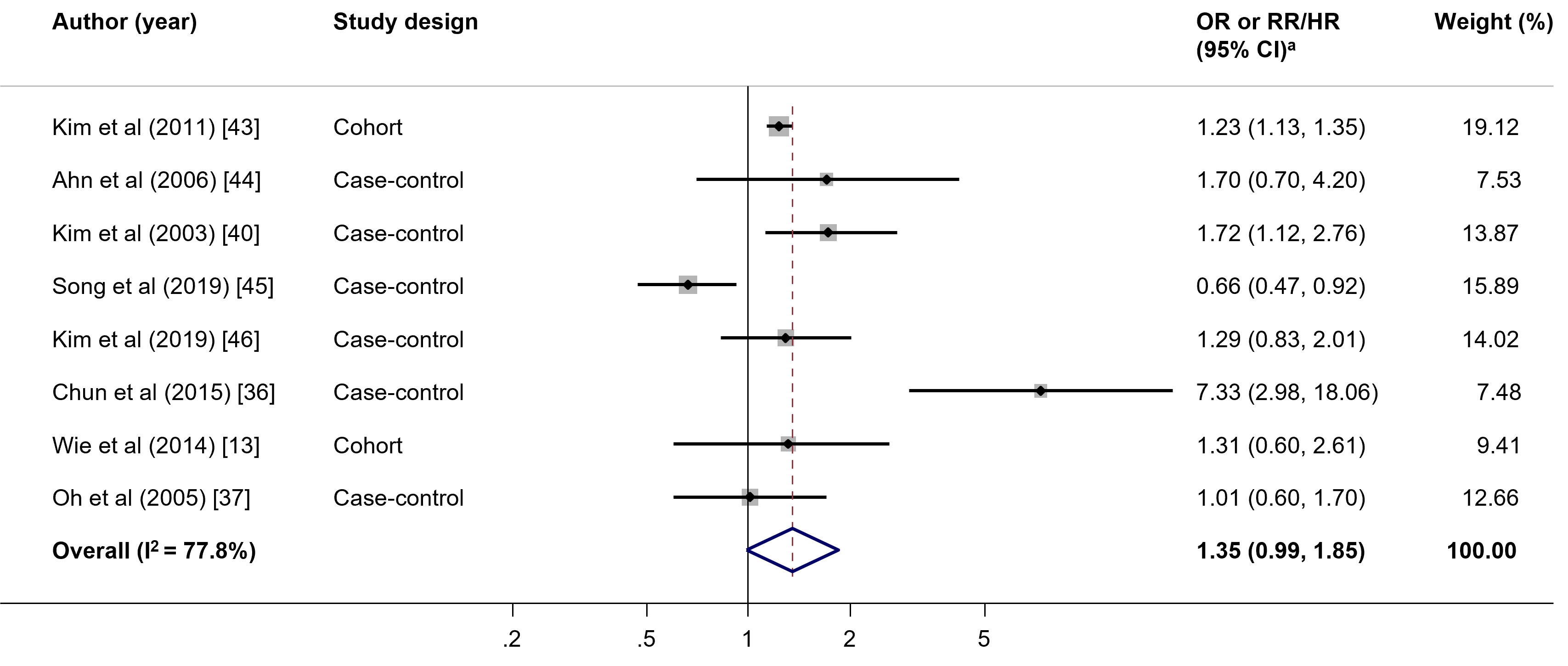

Supplement: Supplement Material 25-1. — Association between meat intake and the risk of colorectal cancer in a random-effects model meta-analysis of observational studies (n=8) [file epih-45-e2023102-Supplementary-25-1.docx]
