# Supplementary material for: Dietary intake and cancer incidence in Korean adults: a systematic review and meta-analysis of observational studies
Source: Epidemiol Health. 2023 Nov 30;45:e2023102. doi: 10.4178/epih.e2023102 (PMC10876448; doi:10.4178/epih.e2023102)

**Supplementary Material 30-2.** Begg’s funnel plot and Egger’s test for identifying publication bias in a meta-analysis of studies on the association between dietary carotenoids intake and the risk of breast cancer (n=4). Each point represents a separate study for the indicated association. SE, standard error; OR, odds ratio.


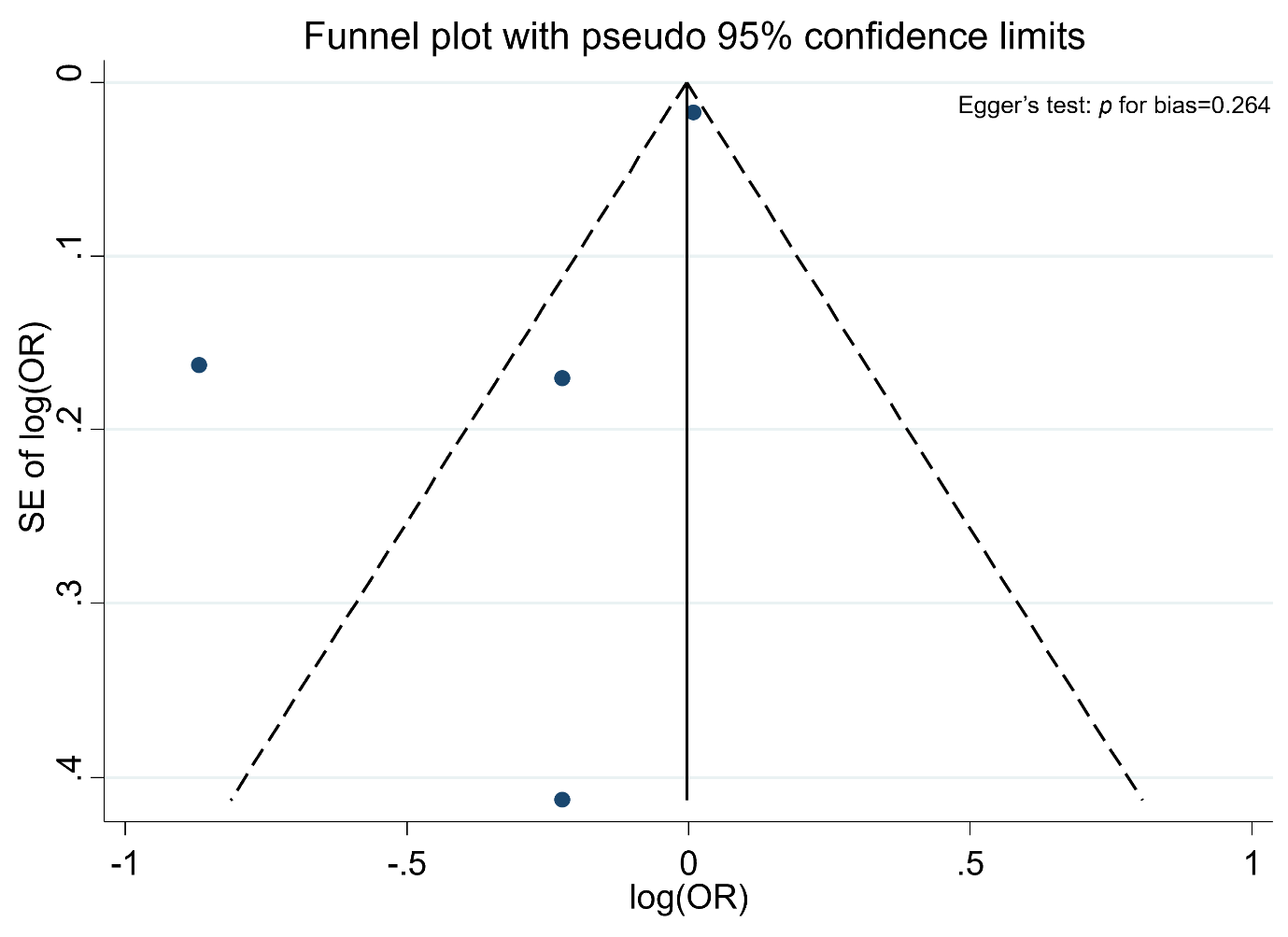

Supplement: Supplement Material 30-2. — Begg’s funnel plot and Egger’s test for identifying publication bias in a meta-analysis of studies on the association between dietary carotenoids intake and the risk of breast cancer (n=4) [file epih-45-e2023102-Supplementary-30-2.docx]
